# Growth Hormone Effects on Hypoxia-Induced Neuroinflammation in the Developing Cerebellum

**DOI:** 10.3390/ijms262110671

**Published:** 2025-11-01

**Authors:** Rosario Baltazar-Lara, Martha Carranza, Carlos G. Martínez-Moreno, José Ávila-Mendoza, Carlos Arámburo, Maricela Luna

**Affiliations:** Laboratorio de Bioquímica de Hormonas, Departamento de Neurobiología Celular y Molecular, Instituto de Neurobiología, Campus Juriquilla, Universidad Nacional Autónoma de México, Querétaro 76230, Mexico; mabal92@comunidad.unam.mx (R.B.-L.); macasa@unam.mx (M.C.); cgmartin@comunidad.unam.mx (C.G.M.-M.); javila@comunidad.unam.mx (J.Á.-M.)

**Keywords:** hypoxia, growth hormone, GH, IGF-1, cerebellum, neuroinflammation, gliosis, neuroprotection

## Abstract

The central nervous system is highly vulnerable to oxygen deprivation during the neonatal period, leading to long-term neurological damage. Growth hormone (GH) has shown neuroprotective and neuroregenerative effects in response to hypoxic injury. This study investigated GH effects on cell survival, inflammatory, and glial activation markers in the developing cerebellum, as well as its impact on motor coordination and anxiety-like behaviors in adulthood following neonatal hypoxia. Global hypoxia was induced in postnatal day 2 Wistar rats (8% O_2_, 2 h), followed by subcutaneous GH treatment (0.1 mg/kg/d) for five days. Neonatal hypoxia triggered a sustained inflammatory response in the developing cerebellum, with increased expression of TLR-4, IL-1β, TNF-α, IL-6, COX-2, iNOS, and pNF-κB, persistent gliosis, myelin disruption, and Purkinje cell loss, leading to impaired adult behavior. GH exhibited a biphasic effect—initially proinflammatory, then anti-inflammatory—ultimately downregulating proinflammatory markers and activating prosurvival pathways (pStat5, pErk1/2, pAkt, Bcl-2, TNF-R2, IGF-1). GH also reduced microglial (Iba-1) and astrocytic (GFAP) hypertrophy, restored MBP and β-III tubulin levels, enhanced Purkinje cell survival, and improved motor coordination and anxiety-like behavior in adulthood. These findings demonstrate that GH modulates the cerebellar inflammatory response and supports its therapeutic potential to counteract neuroinflammation and dysfunction following neonatal hypoxic injury.

## 1. Introduction

The cerebellum is an essential brain structure which coordinates and fine-tunes motor activity in vertebrates. It contains over half of the brain neurons, indicating a critical role in neural processing [1,2]. Recent findings also show that it plays a role in cognition, behavior, and psychiatric disorders [3,4]. In humans, several studies have underscored the vulnerability of the developing cerebellum to hypoxia, particularly during the third trimester of pregnancy and the first month after birth, when its size nearly quadruples [5,6,7,8,9]. This dynamic stage involves continued Purkinje cell differentiation and accelerated proliferation and migration of granule neurons [1,2]. Animal studies in monkeys [10], rats [11], rabbits [12], and chickens [13], demonstrated that perinatal hypoxia causes drastic cerebellar injury and results in important functional alterations.

According to the United Nations International Children’s Emergency Fund (UNICEF) [14], perinatal asphyxia (PA) is the third leading cause (12%) of child mortality worldwide, accounting for nearly 300,000 newborn deaths annually. PA triggers a complex cascade of events that result in brain injury, causing neurodevelopmental impairments with immediate and long-term consequences, such as motor deficits and cognitive and behavioral disorders [15,16]. Following hypoxia, brain injury progresses through several distinctive phases—acute, latent, secondary, and tertiary (chronic)—each marked by alterations in energy metabolism, perfusion, neurochemistry, and inflammatory balance [16]. The main mechanisms underlying this process include energy failure, oxidative stress, intracellular Ca^2+^ accumulation, mitochondrial dysfunction, excitotoxicity, and inflammation [16]. Activation of glial cells—astrocytes and microglia—is an early hallmark of hypoxic brain injury, leading to neuronal and oligodendrocyte loss within hours to days [16,17,18]. Both glial populations display a dual, stage-dependent response: initially amplifying excitotoxic and inflammatory cascades, but later promoting recovery by clearing debris, regulating glutamate uptake, supporting neurovascular coupling, and releasing neurotrophic factors that facilitate neuronal repair [16,17,18]. PA also disrupts the blood–brain barrier, causes hypomyelination, and alters cortical and cerebellar development over months and years [16,17]. Moreover, newborns affected by PA exhibit activation of the innate immune system, evidenced by elevated levels of circulating cytokines [19].

Cerebellar inflammation caused by hypoxic injury (HI) leads to marked microglial activation and increased expression of inflammatory mediators including nuclear factor kappa B (NF-κB), toll-like receptor 4 (TLR4), interleukin-1 beta (IL-1β), tumor necrosis factor alpha (TNF-α), and their receptors [20,21]. Likewise, IL-1 receptor type I (IL-1R1) and TNF receptor 1 (TNFR1), expressed on Purkinje neurons, have been associated with their apoptotic degeneration [21]. These processes were also observed in a model of acute cerebellar inflammation induced by exposure to lipopolysaccharide (LPS), where rodents showed depressive-like behaviors and increased Purkinje neuron excitability—an effect attributed to microglial activation and TNF-α secretion [4]. Microglia are key for synaptic remodeling and Purkinje cell layer refinement during postnatal neurodevelopment [22]; thus, disruptions in their homeostatic roles can impair cerebellar circuit maturation. Chronic cerebellar microglial activation has also been implicated in several neurodevelopmental and neuropsychiatric disorders, including autism spectrum disorder [23].

Growth hormone (GH) has demonstrated strong neuroprotective potential in various neural damage models, including experimental traumatic brain injury [24], spinal cord injury [25], full sciatic nerve lesion [26], cognitive impairment [27], and hypoxia–ischemia [13,28,29]. In the cerebellum, GH exerts neuroprotective effects against HI through molecular and cellular mechanisms that involve neurogenesis, neural migration and maturation, synaptogenesis, neural plasticity, and angiogenic, anti-apoptotic and anti-inflammatory properties both in vivo and in vitro [13,24,25,26,27,28,29,30]. However, current data on the effects of GH upon neuroinflammation and long-term cerebellar functions following hypoxia are limited.

This study aimed to evaluate whether systemic administration of GH during the neonatal period, following HI in newborn rats, could mitigate cerebellar damage, reduce neuroinflammation processes and promote neuroprotection in the developing cerebellum and functional recovery in adulthood.

## 2. Results

### 2.1. Effects of bGH s.c. Injection on GHR mRNA Expression; Activation of pStat5, pErk1/2, pAkt Pathways; And IGF-1 Levels in Neonatal Serum, Cerebellum, and Liver over Time

**Figure 1:**
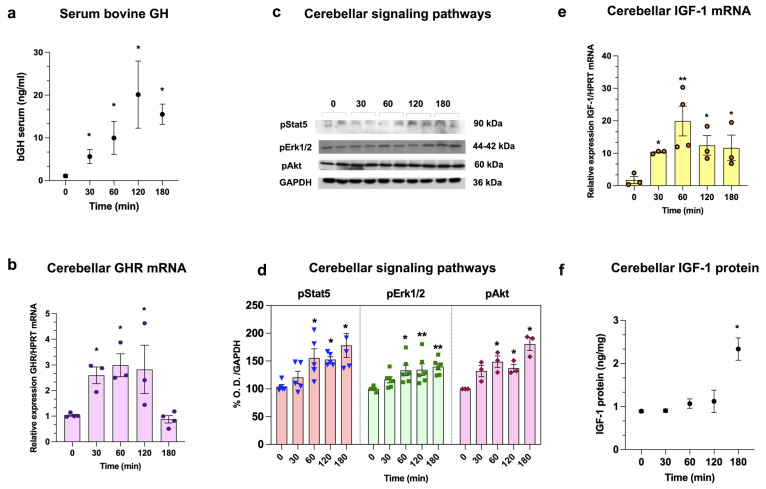
Bovine GH (bGH) administration enhances GHR mRNA, pStat5, pErk1/2, pAkt pathways, and IGF-1 levels in neonatal serum and cerebellum over time. bGH concentration in serum (**a**), GHR mRNA expression (**b**), activation of pStat5, pErk1/2, and pAkt signaling pathways (**c**,**d**), as well as IGF-1 mRNA expression and IGF-1 protein concentration in the cerebellum (**e**,**f**), were determined at 0, 30, 60, 120, and 180 min after a subcutaneous injection of bGH (0.1 mg/kg) on postnatal day 2 (P2). (**a**) Serum concentrations of bGH (*n* = 3/group). (**b**) Relative gene expression of GHR mRNA (*n* = 3/group). (**c**) WB of pStat5 (90 kDa, *n* = 5/group), pErk1/2 (44−42 kDa, *n* = 5/group), pAkt (60 kDa, *n* = 3/group), and GAPDH (36 kDa) as loading control. (**d**) Densitometric analysis of WB. (**e**) Relative gene expression of IGF-1 mRNA. (**f**) Cerebellar tissue concentration of IGF-1(*n* = 3/group). Hypoxanthine phosphoribosyl-transferase (HPRT) was used as the housekeeping gene. Results are shown as mean ± SEM. Asterisks indicate significant differences compared with control (0 min), determined (**a**,**b**,**d**,**e**) by one-way ANOVA with Dunnett post hoc test (* *p* < 0.05; ** *p* < 0.01) and (**f**) by Kruskal–Wallis test.

Before starting HI experiments, a study to determine serum bGH concentration kinetics was conducted at 0, 30, 60, 120, or 180 min after a single bGH dose (0.1 mg/kg/s.c.) was given to control rats on P2 (Figure 1a). A constant and significant rise in bGH concentration was found at 30 min (5.6 ± 1.6 ng/mL, *p* < 0.04) and 60 min (9.9 ± 3.8 ng/mL, *p* < 0.03), which peaked at 120 min (20.1 ± 7.8 ng/mL, *p* < 0.02), and then slightly decreased at 180 min (15.4 ± 2.3 ng/mL, *p* < 0.05) in comparison to the controls (1.09 ± 0.6 ng/mL). In turn, cerebellar GHR mRNA expression was upregulated in response to bGH treatment over time, with increases at 30 min (2.6 ± 0.3-fold, *p* < 0.05), 60 min (2.9 ± 0.4-fold, *p* < 0.05), and 120 min (2.8 ± 0.9-fold, *p* < 0.02), as compared to the controls (1.03 ± 0.1-fold). However, after 180 min, values were similar to the controls (0.8 ± 0.1-fold; Figure 1b).

We used WB to study the time-course activation of GH signaling pathways (pStat5, pErk1/2, and pAkt) in the cerebellum after bGH treatment. Immunoreactivity (IR) bands for pStat5 (90 kDa), pErk1/2 (44–42 kDa), or pAkt (60 kDa) were analyzed by densitometry, using GAPDH (36 kDa) as a loading control (Figure 1c). We found that pStat5-IR increased over time, starting at 60 min (155.3 ± 15.9% *p <* 0.05), and then remained elevated at 120 min (152.6 ± 4.8%, *p* < 0.001) and 180 min (178.1 ± 22%, *p* < 0.05), in comparison to the controls (103.9 ± 12.1%; Figure 1d). Also, pErk1/2-IR showed a sustained increase at 60 min (133.3 ± 9.9%, *p* < 0.05), 120 min (134.6 ± 11.1%, *p* < 0.05), and 180 min (140.1 ± 5.2, *p* < 0.001) in relation to the controls (100.0 ± 7.3%). Similarly, pAkt-IR started to rise at 60 min (148.8 ± 10.3%, *p* < 0.05), continued at 120 min (137.5 ± 6.6%, *p* < 0.01) and further increased at 180 min (180.6 ± 11.4%, *p* < 0.04), as compared to the controls (100.0 ± 13.0%; Figure 1d).

In turn, cerebellar IGF-1 mRNA expression exhibited a bell-shaped curve over time. At 30 and 60 min after the injection of bGH, it rose intensely (10.3 ± 0.3-fold, *p* < 0.05, and 19.9 ± 4.5-fold, *p* < 0.01, respectively), but then started to decline at 120 min (12.4 ± 3.0-fold, *p* < 0.03) and 180 min (11.6 ± 3.9-fold, *p* < 0.05), although it remained higher than the controls (1.7 ± 0.6-fold; Figure 1e). However, the tissue concentration of cerebellar IGF-1 only displayed an increase at 180 min (2.33 ± 0.2 ng/mg, *p* < 0.05), as compared with the control (0.89 ± 0.03 ng/mg; Figure 1f).

On the other hand, in the liver we observed increases in GHR mRNA expression at 30 min (2.1 ± 0.5-fold, *p* < 0.05), 60 min (2.09 ± 0.3-fold, *p* < 0.05), and 120 min (2.4 ± 0.2-fold, *p* < 0.05) following bGH injection, but at 180 min there was no difference with the control (1.1 ± 0.3-fold; Figure S1A). Meanwhile, IGF-1 mRNA expression also increased at 30 min (4.6 ± 1.3-fold, *p* < 0.05), 60 min (3.5 ± 1.0-fold, *p* < 0.05), 120 min (4.3 ± 1.4-fold, *p* < 0.05), and 180 min (3.1 ± 0.3-fold, *p* < 0.05) compared to the control (1.1 ± 0.4-fold; Figure S1B).

### 2.2. Effect of Hypoxia and GH on Inflammatory Factors After Hypoxia Injury and Reoxygenation in the Neonatal Cerebellum

Initially, we studied the acute effects of hypoxia in the cerebellar tissue immediately before the reoxygenation stage at Time = 0 h (acute phase, P2). Therefore, the expression of the transcription factor hypoxia-inducible factor-1 alpha (HIF-1α) mRNA and HIF-1α-IR were analyzed by qPCR and WB, respectively, as a reference to determine the attainment of hypoxic conditions in our experimental model. Results showed that, at time 0 h after HI, HIF-1α mRNA (5.8 ± 1.3-fold, *p* < 0.002; Figure S2A) and HIF-1α-IR (137.9 ± 2.7%, *p* < 0.0001; Figure S2B,C) expression were increased compared to the normoxic controls (0.9 ± 0.8-fold, and 100.0 ± 1.9%, respectively).

**Figure 2:**
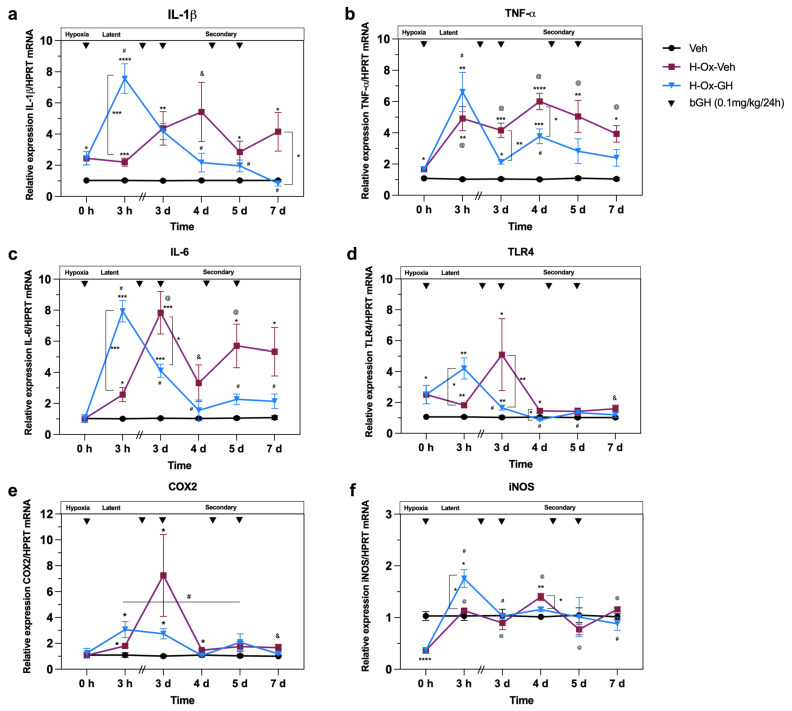
Effects of hypoxia and GH treatment upon inflammatory markers in the neonatal cerebellum following hypoxia-induced injury. Animals were exposed to hypoxic conditions for 2 h on postnatal day 2 (P2). Relative gene expression of (**a**) IL-1β, (**b**) TNF-α, (**c**) IL-6, (**d**) TLR4, (**e**) COX-2 and (**f**) iNOS and mRNA, *n* = 4–8/group, along the four distinct phases of hypoxic injury: acute/hypoxia, latent, early and late secondary phases. Groups: vehicle control (Veh), injured plus reoxygenation (H-Ox-Veh), injured plus reoxygenation plus treated with bovine GH (H-Ox-GH, 0.1 mg/kg/24 h). Results are shown as mean ± SEM. Asterisks indicate significant differences compared with control, determined by Mixed-effects model (REML) with Tukey’s post hoc test (* *p* < 0.05; ** *p* < 0.01; *** *p* < 0.001; **** *p* < 0.0001). Hashtags indicate significant differences in bovine GH (H-Ox-GH) group compared over time [Time = 0 h (acute phase, P2), Time = 3 h (latent phase, P2), Time = 3 d (early secondary phase, P4), Time = 4 d (secondary phase, P5), Time = 5 d (late secondary phase, P6), and Time = 7 d (late secondary phase, P8)] (^#^ *p* < 0.05). At symbols indicate significant differences in the reoxygenation (H-Ox-Veh) group compared over time (^@^ *p* < 0.05). Student’s *t*-test is indicated by the & symbol (^&^ *p* < 0.05).

At Time = 0 h (acute phase, P2), we analyzed the effects of hypoxia upon the mRNA expression of several proinflammatory factors (IL-1β, TNF-α, IL-6, TLR4, COX-2, and iNOS) in the cerebellar tissue. We observed increases in the mRNA expression of IL-1β (2.4 ± 0.4-fold, *p* < 0.051; Figure 2a), TNF-α (1.7 ± 0.1-fold, *p* < 0.05; Figure 2b), and TLR4 (2.5 ± 0.5-fold, *p* < 0.05; Figure 2d), but IL-6 (Figure 2c) and COX-2 (Figure 2e) remained unchanged. In contrast, iNOS expression decreased (0.3 ± 0.08-fold, *p* < 0.0001; Figure 2f) in the HI group.

At Time = 3 h (latent phase, P2), when compared with the corresponding vehicle controls, we found that reoxygenation alone (H-Ox-Veh) induced an increase in the mRNA expression of IL-1β (2.2 ± 0.2-fold, *p* < 0.001; Figure 2a), TNF-α (4.9 ± 0.7-fold, *p* < 0.01; Figure 2b), IL-6 (2.5 ± 0.4-fold, *p* < 0.05; Figure 2c), TLR4 (1.8 ± 0.1-fold, *p* < 0.05; Figure 2d), and COX-2 (1.8 ± 0.1-fold, *p* < 0.05; Figure 2e), while iNOS remained unchanged. Remarkably, bGH treatment (H-Ox-GH) stimulated an increase in IL-1β (7.5 ± 0.9-fold, *p* < 0.001; Figure 2a), IL-6 (7.9 ± 0.6-fold, *p* < 0.001; Figure 2c), TLR4 (4.2 ± 0.6-fold, *p* < 0.05; Figure 2d), and iNOS (1.8 ± 0.1-fold, *p* < 0.05; Figure 2f) mRNA expression compared with vehicle and H-Ox-Veh groups. Furthermore, bGH also enhanced the mRNA expression response of TNF-α (6.6 ± 1.2-fold, *p* < 0.01; Figure 2b) and COX-2 (3.1 ± 0.6-fold, *p* < 0.05; Figure 2e) observed in the H-Ox-Veh group, although in the case of TNF-α and COX-2, this increase was only statistically significant compared with the vehicle.

At Time = 3 d (early secondary phase, P4), the H-Ox-Veh group had significantly higher mRNA levels of IL-1β (4.3 ± 1.1-fold, *p* < 0.05; Figure 2a), TNF-α (4.1 ± 0.4-fold, *p* < 0.01; Figure 2b), IL-6 (7.8 ± 1.3-fold, *p* < 0.05; Figure 2c), TLR4 (5.1 ± 2.3-fold, *p* < 0.05; Figure 2d), and COX-2 (7.2 ± 3.1-fold, *p* < 0.05; Figure 2e) than vehicle controls, while iNOS did not differ significantly. In contrast, bGH reduced TNF-α (2.1 ± 0.1-fold, *p* < 0.05; Figure 2b), IL-6 (4.1 ± 0.4-fold, *p* < 0.05; Figure 2c), TLR4 (1.6 ± 0.1-fold, *p* < 0.05; Figure 2d), and COX-2 (2.7 ± 0.3-fold, *p* < 0.05; Figure 2e) expression in the H-Ox-GH group but did not alter IL-1β (4.1 ± 0.5-fold, *p* < 0.05; Figure 2a). On the other hand, iNOS remained unchanged across groups at this time point.

At Time = 4 d (secondary phase, P5), in the H-Ox-Veh group, IL-1β (5.4 ± 1.9-fold, *p* < 0.02; Figure 2a), TNF-α (6.0 ± 0.5-fold, *p* < 0.0001; Figure 2b), IL-6 (3.3 ± 1.1-fold, *p* < 0.05; Figure 2c), TLR4 (1.5 ± 0.1-fold, *p* < 0.05; Figure 2d), COX-2 (1.5 ± 0.07-fold, *p* < 0.05; Figure 2e), and iNOS (1.4 ± 0.07-fold, *p* < 0.01; Figure 2f) were increased in relation to the control. Meanwhile, H-Ox-GH reduced TNF-α (3.7 ± 0.4-fold, *p* < 0.001; Figure 2b), TLR4 (0.8 ± 0.04-fold, *p* < 0.01; Figure 2d), and iNOS (1.2 ± 0.07-fold, *p* < 0.05; Figure 2f) compared with H-Ox-Veh, while IL-1β, IL-6, and COX-2 returned to levels similar to those of the normoxic control.

At Time = 5 d (late secondary phase, P6), the H-Ox-Veh group showed increases in IL-1β (2.8 ± 0.7-fold, *p* < 0.05; Figure 2a), TNF-α (5.0 ± 1.0-fold, *p* < 0.001; Figure 2b), and IL-6 (5.7 ± 1.4-fold, *p* < 0.05; Figure 2c). However, bGH treatment did not alter IL-1β, TNF-α, IL-6, TLR4, COX-2, or iNOS, resulting in expression levels similar to those in vehicle controls.

Finally, at Time = 7 d (late secondary phase, P8), IL-1β (4.1 ± 1.2-fold, *p* < 0.05; Figure 2a), TNF-α (2.3 ± 0.5-fold, *p* < 0.05; Figure 2b), IL-6 (2.13 ± 0.4-fold, *p* < 0.05; Figure 2c), TLR4 (1.6 ± 0.2-fold, *p* < 0.05; Figure 2d), and COX-2 (1.7 ± 0.2-fold, *p* < 0.05; Figure 2e) were all elevated in the H-Ox-Veh group compared with vehicle controls. In contrast, bGH reversed the increase in IL-1β (0.8 ± 0.1-fold, *p* < 0.05; Figure 2a) and normalized TNF-α (1.2 ± 0.1-fold, *p* < 0.05; Figure 2b) and IL-6 (1.3 ± 0.2-fold, *p* < 0.001; Figure 2c) to levels similar to those of the normoxic control. COX-2 and iNOS showed no changes in any group at this time point.

### 2.3. Role of GH Treatment in Modulating Signaling Pathways, Anti-Apoptotic and Inflammatory Markers During the Latent and Secondary Phases of Hypoxic Injury in the Neonatal Cerebellum

In addition, we evaluated the impact of HI and subsequent treatments on various signaling pathways, as well as on anti-apoptotic and inflammatory markers that may mediate the neuroprotective effects of GH. These analyses were performed by WB, using GAPDH as a loading control (Figure S3).

**Figure 3:**
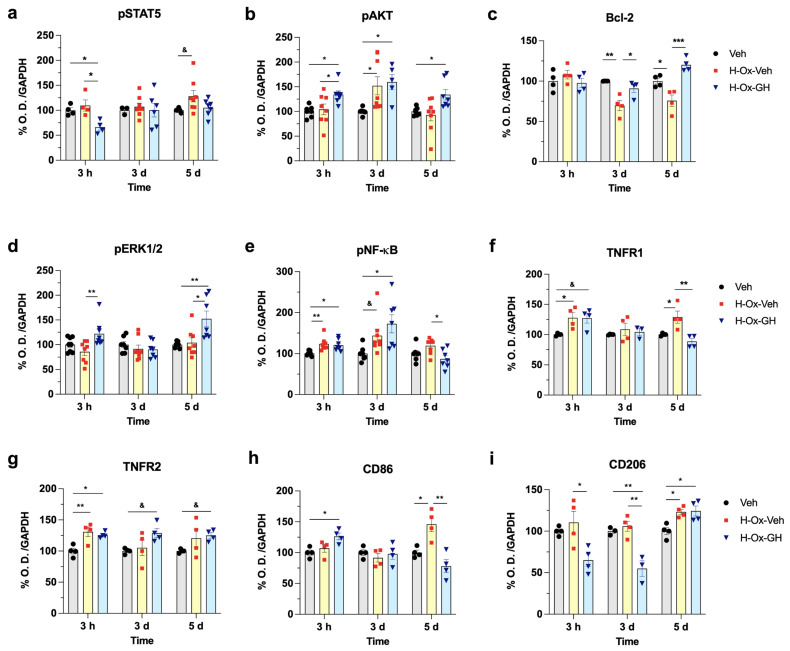
Effects of hypoxia and GH treatment upon the activation of signaling pathways, anti-apoptotic and inflammatory markers in the neonatal cerebellum following hypoxia-induced injury. Animals were exposed to hypoxic conditions for 2 h on postnatal day 2 (P2). Densitometric analysis of Western blotting (Figure S3) of (**a**) pStat5, (**b**) pAkt, (**c**) Bcl-2, (**d**) pErk1/2, (**e**) pNF-κB, (**f**) TNF-R1, (**g**) TNF-R2, (**h**) CD86, (**i**) CD206 factors, GAPDH as loading control, *n* = 3–8/group, along the several phases of hypoxic injury: latent, early and late secondary phases. Groups: Vehicle control (Veh), injured plus Reoxygenation (H-Ox-Veh), injured plus reoxygenation plus treated with bovine GH (H-Ox-GH, 0.1 mg/kg/24 h). Results are shown as mean ± SEM. Asterisks indicate significant differences compared with control (vehicle), determined by one-way ANOVA with Tukey’s post hoc test (* *p* < 0.05; ** *p* < 0.01; *** *p* < 0.001). Student’s *t*-test is indicated by the & symbol (^&^ *p* < 0.05).

Figure 3 shows cerebellar changes in the IR of pStat5 (Figure 3a), pAkt (Figure 3b), Bcl-2 (Figure 3c), pErk1/2 (Figure 3d), pNF-κB (Figure 3e), TNFR1(Figure 3f), TNFR2 (Figure 3g), CD86 (Figure 3h), and CD206 (Figure 3i).

Results at Time = 3 h (latent phase, P2) indicated that, compared to the vehicle (~100%), the H-Ox-Veh group exhibited increases in pNF-κB-IR (123.2 ± 5.5%, *p* < 0.01; Figure 3e), TNFR1-IR (127.8 ± 8.5%, *p* < 0.05; Figure 3f), and TNFR2-IR (130.6 ± 7.6%, *p* < 0.01; Figure 3g). In turn, bGH treatment also stimulated a marked increase in pAkt-IR (134.3 ± 10.8%, *p* < 0.05; Figure 3b), pNF-κB-IR (120.0 ± 4.6%, *p* < 0.05; Figure 3e), TNFR1-IR (127.0 ± 8.2%, *p* < 0.05; Figure 3f), TNFR2-IR (125.6 ± 2.5%, *p* < 0.05; Figure 3g), and CD86-IR (126.3 ± 5.4%, *p* < 0.05; Figure 3h) as compared to the controls. In contrast, bGH induced a reduction in pStat5-IR (66.4 ± 6.4%, *p* < 0.05; Figure 3a) and CD206-IR (65.1 ± 7.6%, *p* < 0.05; Figure 3i) in relation to the vehicle group. On the other hand, no differences in Bcl-2-IR were observed between groups, while bGH increased pErk1/2-IR (121.9.0 ± 9.3%, *p* < 0.01; Figure 3d) in comparison to the H-Ox-Veh group, but not the vehicle control.

At Time = 3 d (early secondary phase, P4), an increase in pAkt-IR (151.9 ± 18.3%, *p* < 0.05; Figure 3b) and pNF-κB-IR (143.5 ± 15.9%, *p* < 0.05; Figure 3e) was observed in the H-Ox-Veh group, while the Bcl-2-IR (69.7 ± 6.2%, *p* < 0.01; Figure 3c) was reduced compared to the vehicle group.

Similarly to the latent phase, bGH treatment stimulated an essential rise in pAkt-IR (159.6 ± 15.3%, *p* < 0.05; Figure 3b), pNF-κB-IR (172.6 ± 22.2%, *p* < 0.05; Figure 3f), and TNFR2-IR (128. ± 7.3%, *p* < 0.05; Figure 3g) as compared to the controls. Interestingly, bGH induced an increase in Bcl-2-IR (90.8 ± 4.9%, *p* < 0.05; Figure 3c) in relation to the H-Ox-Veh group. In contrast, bGH decreased CD206-IR (124.3 ± 6.0%, *p* < 0.01; Figure 3i) compared with the H-Ox-Veh group and control.

At Time 5 = d (late secondary phase, P6), HI exhibited elevations in pStat5-IR (128.1 ± 11.4%, *p* < 0.05; Figure 3b), TNFR1-IR (128.8 ± 10.0%, *p* < 0.05; Figure 3f), CD86-IR (145.9 ± 11.8%, *p* < 0.05; Figure 3h), and CD206-IR (123.1 ± 3.0%, *p* < 0.05; Figure 3i) compared with the vehicle. Similarly, at Time = 5 d (late secondary phase, P6), the Bcl-2-IR (75.6 ± 7.1%, *p* < 0.05; Figure 3c) was reduced in the H-Ox-Veh group. The signaling pathways involved in GH actions showed that pAkt-IR (134.0 ± 10.8%, *p* < 0.05; Figure 3b), Bcl-2 (120.1 ± 4.2%, *p* < 0.001; Figure 3c), pErk1/2-IR (152.1 ± 15.8%, *p* < 0.01; Figure 3d), TNFR2-IR (125.0 ± 4.4%, *p* < 0.05; Figure 3g), and CD206-IR (124.3 ± 6.0%, *p* < 0.05; Figure 3i) increased with bGH treatment, compared to the vehicle group. Conversely, pNF-κB-IR (87.0 ± 8.5%, *p* < 0.05; Figure 3e), TNFR1-IR (88.5 ± 4.9%, *p* < 0.01; Figure 3f), and CD86-IR (78.1 ± 10.5%, *p* < 0.01; Figure 3h) decreased in the H-Ox-GH group relative to the vehicle control.

### 2.4. Hypoxia and GH Effects on Growth Factors During the Acute, Latent and Secondary Phases of the Hypoxic Injury in the Neonatal Cerebellum

**Figure 4:**
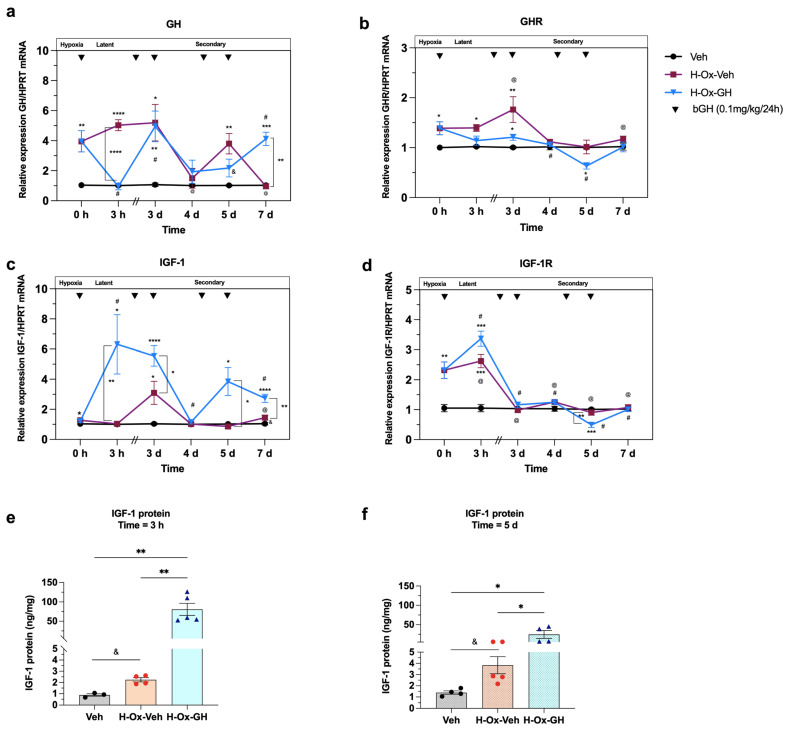
Effects of hypoxia and GH treatment upon GH/IGF-1 system expression after a hypoxia-induced injury in the neonatal cerebellum. Animals were exposed to hypoxic conditions for 2 h on postnatal day 2 (P2). Relative mRNA expression of (**a**) GH, (**b**) GHR, (**c**) IGF-1, and (**d**) IGF-1R, as determined by qPCR. Hypoxanthine phosphoribosyl-transferase (HPRT) was used as the housekeeping gene. *n* = 4–8/group along several phases of hypoxic injury: acute/hypoxia, latent, early and late secondary phases. Cerebellar tissue concentrations of IGF-1 after (**e**) one bGH dose (Time = 3 h, latent phase, P2) and (**f**) five bGH doses per day (Time = 5 d, late secondary phase, P6), *n* = 3–5/group. Groups: Vehicle control (Veh), injured plus Reoxygenation (H-Ox-Veh), injured plus reoxygenation plus treated with bovine GH (H-Ox-GH, 0.1 mg/kg/24h). Results are shown as mean ± SEM. Asterisks indicate significant differences compared with control, determined by one way ANOVA and Mixed-effects model (REML) with Tukey’s post hoc test (* *p* < 0.05; ** *p* < 0.01; *** *p* < 0.001; **** *p* < 0.0001). Hashtags indicate significant differences in bovine GH (H-Ox-GH) group compared over time [Time = 0 h (acute phase, P2), Time = 3 h (latent phase, P2), Time = 3 d (early secondary phase, P4), Time = 4 d (secondary phase, P5), Time = 5 d (late secondary phase, P6), and Time = 7 d (late secondary phase, P8)](^#^ *p* < 0.05). At symbols indicate significant differences in reoxygenation (H-Ox-Veh) group compared over time (^@^ *p* < 0.05). Student’s *t*-test is indicated by the & symbol (^&^ *p* < 0.05).

We also evaluated how HI affects cerebellar expression of GH, GHR, IGF-1, and IGF-1R mRNAs. At Time = 0 h (acute phase, P2), HI led to higher mRNA levels of GH (3.9 ± 0.7-fold, *p* < 0.0002; Figure 4a), GHR (1.4 ± 0.1-fold, *p* < 0.0002; Figure 4b), IGF-1 (1.3 ± 0.07-fold, *p* < 0.03; Figure 4c), and IGF-1R (2.3 ± 0.2-fold, *p* < 0.05; Figure 4d) compared with the normoxic group (~1.0-fold).

Furthermore, at Time = 3 h (latent phase, P2), the mRNA expression of GH (5.0 ± 0.3-fold, *p* < 0.0003; Figure 4a), GHR (1.4 ± 0.06-fold, *p* < 0.05; Figure 4b), and IGF-1R (2.6 ± 0.2-fold, *p* < 0.05, Figure 4d) was increased in the H-Ox-Veh group, while IGF-1 (Figure 4c) mRNA showed no changes in comparison to the vehicle group. Notably, a single dose of bGH induced an increase in IGF-1 (6.3 ± 1.9-fold, *p* < 0.0001; Figure 4b) mRNA expression compared to the vehicle and H-Ox-Veh groups. Also, bGH raised IGF-1R mRNA (3.3 ± 0.2-fold, *p* < 0.0001; Figure 4d) relative to the vehicle control, whereas GHR mRNA remained unchanged in comparison to both the vehicle and H-Ox-Veh. Interestingly, bGH decreased local GH mRNA expression (0.9 ± 0.2-fold, *p* < 0.05; Figure 4a) to levels similar to those of the vehicle control. In addition, IGF-1 concentration in cerebellar tissues was determined by ELISA in the three groups. Results showed that bGH treatment induced an increase (80.7 ± 15.6 ng/mg, *p* < 0.01) in IGF-1 as compared to the H-Ox-Veh (2.2 ± 0.1 ng/mg) and vehicle (0.9 ± 0.1 ng/mg) groups, Figure 4e.

At Time = 3 d (early secondary phase, P4), H-Ox-Veh group showed higher mRNA levels of GH (5.2 ± 1.2-fold, *p* < 0.01; Figure 4a), GHR (1.8 ± 0.2-fold, *p* < 0.05; Figure 4b), and IGF-1 (3.0 ± 0.7-fold, *p* < 0.002; Figure 4c) mRNA expression compared with the vehicle control, while IGF-1R (Figure 4d) remained unchanged. bGH treatment further stimulated IGF-1 mRNA (5.5 ± 0.6-fold, *p* < 0.0001; Figure 4c) compared with both the vehicle and H-Ox-Veh groups. In addition, bGH increased the expression of GH (4.9 ± 1.0-fold, *p* < 0.007; Figure 4a) and GHR (1.2 ± 0.06-fold, *p* < 0.007; Figure 4b) mRNAs as compared with the vehicle control, with no changes in IGF-1R (Figure 4d) in either group, Figure 4.

At Time = 4 d (secondary phase, P5), no changes were observed in the mRNA expression of GH, GHR, IGF-1, and IGF-1R in either group (Figure 4a–d).

At Time = 5 d (late secondary phase, P6), IGF-1 (3.8 ± 0.9-fold, *p* < 0.0001; Figure 4c) mRNA expression was higher in the H-Ox-GH group than in both vehicle and H-Ox-GH groups, whereas GHR (0.6 ± 0.06-fold, *p* < 0.05; Figure 4b) and IGF-1R (0.4 ± 0.08-fold, *p* < 0.05; Figure 4d) mRNAs were markedly reduced relative to the vehicle control. Notably, GH mRNA (3.8 ± 0.6-fold, *p* < 0.05; Figure 4a) was also elevated in the H-Ox-Veh group compared with the vehicle. At this stage, IGF-1 concentration in cerebellar tissue (Figure 4f) was increased in the H-Ox-GH group (24.6 ± 10.26 ng/mg, *p* < 0.05) compared with both the vehicle (1.4 ± 0.1 ng/mg) and H-Ox-Veh (3.8 ± 0.7 ng/mg) groups. Also, IGF-1 was higher (*p* < 0.05) in the H-Ox-Veh group relative to the normoxic control.

Finally, at Time = 7 d (late secondary phase, P8), we found that GH (4.1 ± 0.4-fold, *p* < 0.01; Figure 4a) and IGF-1 (2.7 ± 0.2-fold, *p* < 0.01; Figure 4c) mRNA levels were higher in the bGH-treated group than in both the vehicle and H-Ox-Veh groups. In contrast, GHR and IGF-1R mRNA expression remained unchanged across all groups. Notably, in the H-Ox-Veh group, only IGF-1 mRNA (1.4 ± 0.1-fold, *p* < 0.01) was elevated relative to vehicle control (Figure 4c), while no differences were observed in the other markers. Figure S4 presents an illustrative summary of the effects of GH on inflammatory markers, apoptotic regulators, intracellular signaling pathways, and growth factors in the neonatal cerebellum following global hypoxia.

### 2.5. GH Effects upon Microglial Morphological Changes in the Adult Cerebellum After Neonatal HI

**Figure 5:**
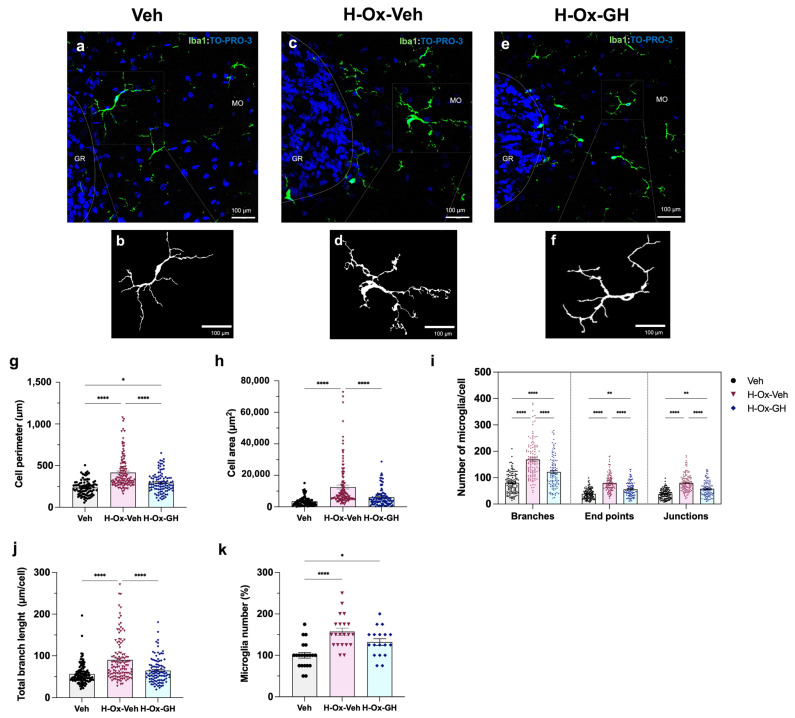
GH prevents microglial reactivity following hypoxic injury in the adult cerebellum. Representative photomicrographs of Iba-1-stained microglia among experimental groups: (**a**) Vehicle (Veh), (**c**) injured plus Reoxygenation (H-Ox-Veh), (**e**) injured plus reoxygenation plus treated with bovine GH (H-Ox-GH, 0.1 mg/kg/24 h). Binary isolated microglia of Veh (**b**), H-Ox-Veh (**d**) and H-Ox-GH (**f**). (**g**) Perimeter of cell bodies (μm). (**h**) Area of microglia cell bodies (μm^2^). (**i**) Number of branches, end points and junctions per microglial cell. (**j**) Total branch length (μm) per microglial cell. (**k**) Microglia cell number percentage (%). Results are expressed as mean ± SEM (*n* = 3 slices per group, twenty representative fields per slice). Asterisks indicate significant differences compared with control, determined by one-way ANOVA with Tukey’s post hoc test (* *p* < 0.05; ** *p* < 0.01; **** *p* < 0.0001). MO, molecular layer; GR, granular layer. Scale bar = 100 µm. Veh: *n* = 138 microglia, H-Ox-Veh: *n* = 180 microglia, H-Ox-GH *n* = 145 microglia.

Immunohistochemical analysis was used to study the long-term effects of bGH treatment upon morphological aspects of cerebellar microglia in adult rats (P63, tertiary phase) that were exposed to HI during the neonatal stage. Figure 5a–f show cerebellar slides that were labeled with a specific antibody against Iba-1 (green) and counterstained with To-Pro3 (blue) as a nuclear dye.

Images were captured using a confocal microscope with 40× magnification, and the morphological parameters of microglia were assessed at two levels: complete photomicrograph analysis (Figure S5A,B) or single-cell evaluation (Figure S5C) utilizing fractal analysis (Figure S5D) and individual skeletal analysis (Figure S5E,F). To ensure unbiased quantification, microglial cell counts were conducted by an independent observer who was blinded to the treatment groups.

Results indicated that microglia from the H-Ox-Veh group showed a larger cell body perimeter (417.3 ± 17.8 µm, *p* < 0.001; Figure 5g) and cell area (12,577 ± 1245 µm^2^, *p* < 0.001; Figure 5h) than those in the vehicle group (236.3 ± 9.7 µm and 3508.9 ± 307.4 µm^2^, respectively). In turn, the bGH treatment group exhibited microglia with a reduced perimeter (295.9 ± 12.6 µm *p* < 0.001; Figure 5g) and area (6149 ± 542.8 µm^2^, *p* < 0.001; Figure 5h) in comparison to the H-Ox-Veh group and showed no differences compared to the normoxic control. Likewise, the lesioned group showed considerable increases in the number of cell branches (169.0 ± 6.2, *p* < 0.0001), endpoints (79.4 ± 2.9, *p* < 0.004), and junctions (80.1 ± 2.9, *p* < 0.001) compared to the control group, Figure 5i. In contrast, the H-Ox-GH group demonstrated a reduction in cell branches (121.6 ± 6.1, *p* < 0.001), endpoints (55.9 ± 2.8, *p* < 0.001), and junctions (57.6 ± 2.8, *p* < 0.001) per cell when compared to the HI group. On the other hand, microglia from the treatment group exhibited a shorter branch length (64.6 ± 2.9 µm, *p* < 0.001) than that of the H-Ox-Veh group (90.3 ± 4.6 µm), but it was not different than the vehicle group (56.3 ± 2.2 µm), Figure 5j. Lastly, Figure 5k shows an increase (137.7 ± 6.6%, *p* < 0.01) in the percentage of microglia in the H-Ox-Veh cerebellum as compared to the normoxic control (100.0 ± 6.8%). However, the bGH (122.3 ± 6.5%) treatment group showed no differences with the other groups.

### 2.6. GH Effects on GFAP and MBP Immunoreactivity in the Adult Cerebellum After a Neonatal Hypoxia-Induced Injury

**Figure 6:**
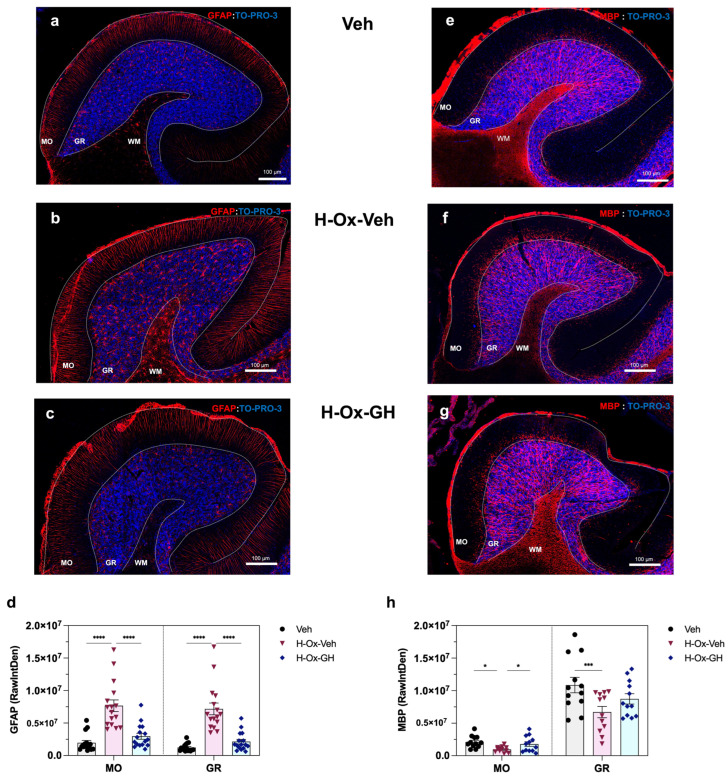
Glial fibrillary acidic protein (GFAP) and myelin basic protein (MBP) immunohistochemistry in adult cerebellum. Parasagittal sections of the cerebellum at 10× magnification. Experimental groups: Vehicle control (Veh; (**a**,**e**)), injured plus Reoxygenation (H-Ox-Veh; (**b**,**f**)), injured plus reoxygenation plus treated with bovine GH (H-Ox-GH; (**c**,**g**)). Red: GFAP (**a**–**c**) or MBP (**e**–**g**) immunofluorescence; Blue: TO-PRO-3 staining (nuclei). (**d**) Integrated density (RawIntDent) of GFAP in MO and GR layers. (**h**) Integrated density of MBP in MO and GR layers. Results are expressed as mean ± SEM (*n* = 4 slices per group, three-five representative fields per slice). Asterisks indicate significant differences compared with control, determined by one-way ANOVA with Tukey’s post hoc test (* *p* < 0.05; *** *p* < 0.001; **** *p* < 0.0001). MO, molecular layer; GR, granular layer; WM, white matter. Scale bar = 100 µm.

Figure 6 shows results in each of the experimental groups, obtained in cerebellar sections from adult rats, that were labeled with specific antibodies directed against either glial fibrillary acidic protein (GFAP, red; Figure 6a–c) or myelin basic protein (MBP, red; Figure 6e–g) and counterstained with To-Pro3 (blue) as a nuclear marker. Compared to the vehicle control (Figure 6a), an increase in GFAP-IR was exhibited in the H-Ox-Veh group, specifically in the molecular (MO) and granular (GR) layers (Figure 6b), with additional prominent staining observed in the white matter (WM). In contrast, the treatment with bGH clearly reversed the effect produced by the injury in all layers (Figure 6c). Quantification of the integrated density (RawIntDen) of GFAP in the corresponding slides confirmed these results. Thus, as shown in Figure 6d, GFAP-IR was higher in the H-Ox-Veh group in both layers (MO: 7.6 ± 0.9 × 10^6^ RawIntDen, *p* < 0.001; and GR: 7.1 ± 0.9 × 10^6^ RawIntDen, *p* < 0.001, respectively) in comparison to the controls (MO: 1.9 ± 0.3 × 10^6^ RawIntDen; GR: 1.2 ± 0.1 × 10^6^ RawIntDen), whereas in the H-Ox-bGH group, GFAP-IR decreased (*p* < 0.001) in MO (2.9 ± 0.45 × 10^6^ RawIntDen) and GR (2.1 ± 0.35 × 10^6^ RawIntDen) layers, reaching values similar to those of the vehicle controls (Figure 6d). Although GFAP-IR was not quantitatively assessed in the WM, a marked increase in the number of GFAP-positive astrocytes was evident in the H-Ox-Veh group (Figure 6b) relative to the vehicle controls (Figure 6a), while GH treatment drastically reduced astrocytic density in this region (Figure 6c).

A marked reduction in MBP-IR was observed in the H-Ox-Veh group (Figure 6f) compared with controls (Figure 6e), whereas bGH treatment effectively reversed this effect (Figure 6g). Quantitative analysis of MBP-IR (*p* < 0.01 for both) in the MO (0.96 ± 0.12 × 10^6^ RawIntDen) and GR (6.7 ± 0.86 × 10^6^ RawIntDen) layers relative to the vehicle group (Figure 6h) corroborated these findings. In the MO layer, bGH administration significantly increased MBP-IR (1.7 ± 0.35 × 10^6^ RawIntDen) compared with the H-Ox-Veh group. However, no significant differences were detected in the GR layer when compared with the vehicle (MO: 2.1 ± 0.2 × 10^6^ RawIntDen; GR: 1.0 ± 0.1 × 10^7^ RawIntDen) or HI groups (Figure 6h). Although MBP-IR in the WM was not quantified, a clear reduction was noted in the H-Ox-Veh group (Figure 6f) compared with vehicle controls (Figure 6e). Importantly, bGH treatment promoted recovery of myelin integrity in this region (Figure 6g).

### 2.7. GH Modulation of CAlbindin and β-Tubulin Immunoreactivity in the Adult Cerebellum After Neonatal Hypoxic Injury

**Figure 7:**
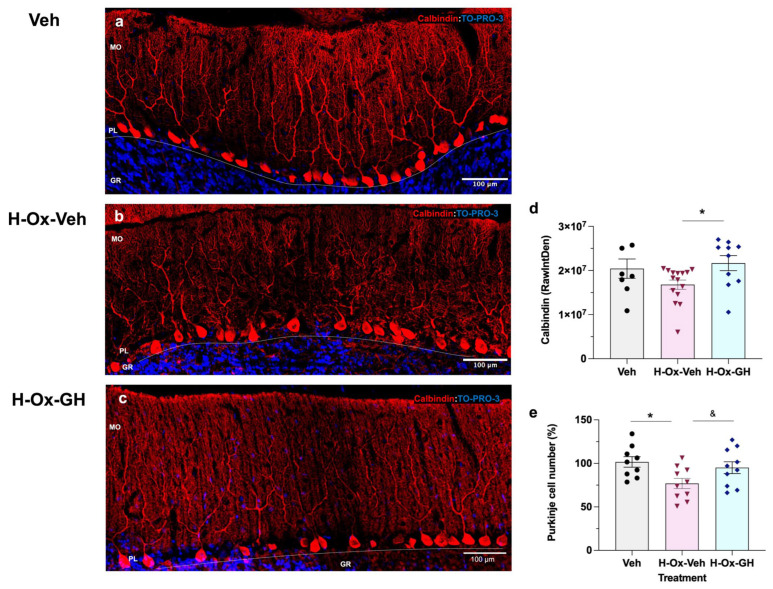
Calbindin immunohistochemistry in adult cerebellum. Parasagittal sections of the cerebellum. Experimental groups: Vehicle control (Veh; (**a**)), injured plus Reoxygenation (H-Ox-Veh; (**b**)), injured plus reoxygenation plus treated with bovine GH (H-Ox-GH; (**c**)). Red: Calbindin immunofluorescence; Blue: TO-PRO-3 staining (nuclei). (**d**) Integrated density (RawIntDent) of Calbindin in PL layer. (**e**) Purkinje cell number percentage (%). Results are expressed as mean ± SEM (*n* = 3 slices per group, four representative fields per slice). Asterisks indicate significant differences compared with control, determined by one-way ANOVA with Tukey’s post hoc test (* *p* < 0.05). Student’s *t*-test is indicated by the & symbol (^&^ *p* < 0.05). MO, molecular layer; PL, Purkinje layer; GR, granular layer; WM, white matter. Scale bar = 100 µm. Veh: *n* = 187 Purkinje, H-Ox-Veh: *n* = 156 Purkinje, H-Ox-GH: *n* = 199 Purkinje.

**Figure 8:**
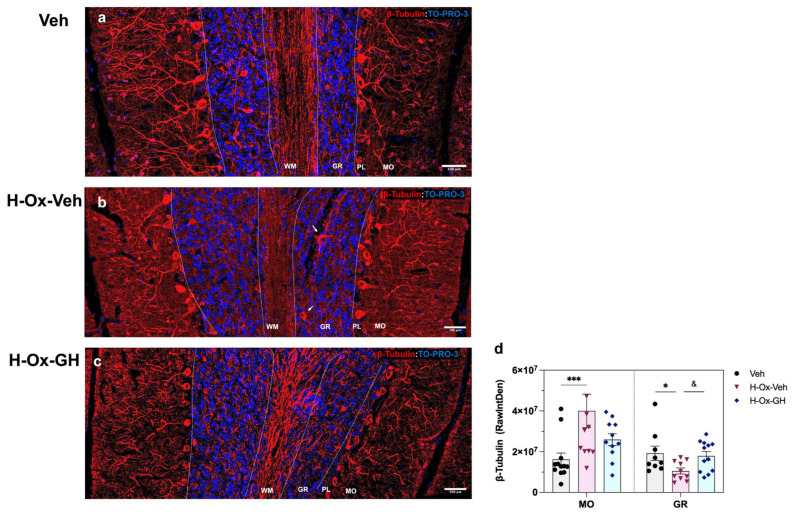
β-Tubulin immunohistochemistry in adult cerebellum. Parasagittal sections of the cerebellum. Experimental groups: Vehicle control (Veh; **a**), injured plus Reoxygenation (H-Ox-Veh; **b**), injured plus reoxygenation plus treated with bovine GH (H-Ox-GH; **c**). Red: β-Tubulin immunofluorescence; Blue: TO-PRO-3 staining (nuclei). (**d**) Integrated density (RawIntDent) of β-Tubulin in MO and GR layer. Results are expressed as mean ± SEM (*n* = 4 slices per group, three representative fields per slice). White arrows indicate ectopic neurons. Asterisks indicate significant differences compared with control, determined by one-way ANOVA with Tukey’s post hoc test (* *p* < 0.05; *** *p* < 0.001). Student’s *t*-test is indicated by the & symbol (^&^ *p* < 0.05). MO, molecular layer; PL, Purkinje layer; GR, granular layer; WM, white matter. Scale bar = 100 µm.

Cerebellar tissues obtained from adult rats in all experimental groups were labeled with specific antibodies directed against either calbindin (red), which specifically labels Purkinje cells (Figure 7), or β-tubulin (red), a marker of mature neurons (Figure 8). Tissues were counterstained with the To-Pro3 (blue) nuclear dye. Figure 7e showed a reduction (77.2 ± 3.6%, *p* < 0.01) in the proportion of Purkinje cells in the HI group compared to the group that received GH treatment (95.6 ± 4.3%) and the vehicle control (100.0 ± 4.6%), while no changes were observed between the GH and vehicle groups. Additionally, measurement of the integrated density of calbindin-IR cells indicated that GH treatment increased (2.1 ± 0.1 × 10^7^ RawIntDen; Figure 7c,d) compared to the HI group (1.6 ± 0.1 × 10^7^ RawIntDen, Figure 7b,d) but not the vehicle control (Figure 7a,d). In addition to the reduction in Purkinje cell number and calbindin signal, a marked disorganization of the dendritic arborization was evident in the H-Ox-Veh group, in contrast to the well-preserved dendritic architecture observed in the vehicle and GH-treated groups.

Figure 8 shows the results obtained with β-tubulin (red) immunostaining. In the MO layer, the hypoxia-lesioned group (Figure 8b,d) exhibited a rise in the integrated density (4.0 ± 0.82 × 10^7^ RawIntDen, *p* < 0.05) of β-tubulin-IR compared to both the vehicle (1.6 ± 0.31 × 10^7^ RawIntDen; Figure 8a,d) and GH treatment (2.5 ± 0.29 × 10^7^ RawIntDen; Figure 8c,d) groups. In contrast, the GR layer in the HI group (Figure 8b,d) showed a clear reduction (1.1 ± 0.14 × 10^7^ RawIntDen, *p* < 0.05) in β-tubulin-IR compared to the vehicle group (1.9 ± 0.39 × 10^7^ RawIntDen; Figure 8a,d). Significant differences (*p* < 0.05) were observed between the GH-treated group and the damage conditions. In addition, the H-Ox-Veh group (Figure 8b) exhibited marked disorganization of Purkinje cell dendritic arborization in the MO layer, accompanied by reduced β-III-tubulin expression in the WM, indicative of cytoskeletal alterations. β-III-tubulin staining also revealed ectopic Purkinje cell bodies with abnormal localization.

### 2.8. Impact of GH on Locomotor Activity, Anxiety-Related Behavior and Motor Coordination in Adult Rats After a Neonatal Hypoxia-Induced Injury

**Figure 9:**
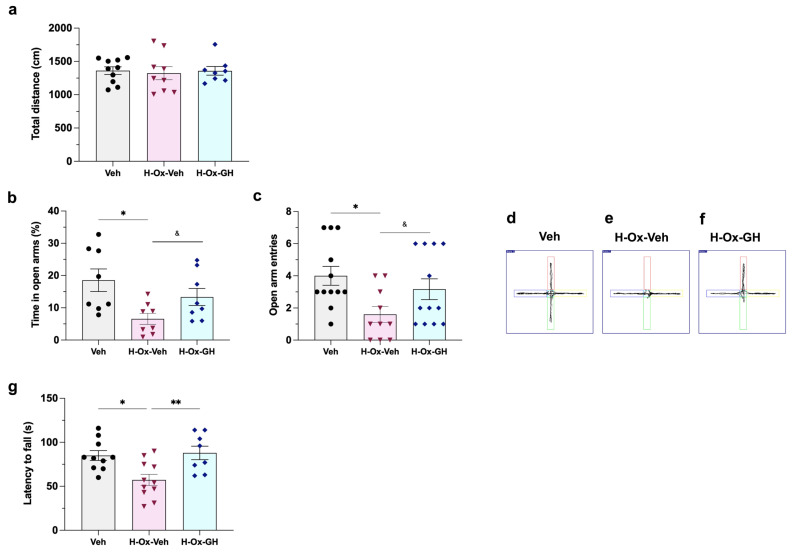
Effects of GH treatment upon locomotor activity, anxiety-related behavior, and motor coordination after a neonatal hypoxia-induced injury in the adult rat. The following behavioral tests were applied to P36 rats: (**a**) Open field locomotor activity; (**b**–**f**) Elevated plus-maze: (**b**) percentage of time spent in open arms and (**c**) number of open arm entries. Route diagram of (**d**) Veh; (**e**) H-Ox-Veh; (**f**) H-Ox-GH on the elevated plus-maze (red and green: open arms; blue and yellow: closed arms). (**g**) In addition, the performance of rats in the rotarod test (latency to fall in seconds) was evaluated at P62. Results are presented as mean ± SEM. *n* = 8–12/group. Asterisks indicate significant differences compared with control, determined by one-way ANOVA with Tukey’s post hoc test (* *p* < 0.05; ** *p* < 0.01). Student’s *t*-test is indicated by the & symbol (^&^ *p* < 0.05).

Locomotor activity was assessed at P36 (tertiary phase) using the open-field test. In this assay the results showed that there were no significant differences between any of the experimental groups (Figure 9a).

On the other hand, the elevated plus-maze test revealed that HI affected the anxiety-related behavior of this group, as evidenced by a clear reduction in the number of entries (1.6 ± 0.4, *p* < 0.05; Figure 9b,e) into the open arms in comparison to the vehicle group (4 ± 0.5 entries; Figure 9b,d). Similarly, when evaluating the time spent exploring in the open arms, rats in the H-Ox-Veh group displayed a decrease (6.5 ± 1.7%, *p* < 0.05; Figure 9c,e) in the time spent in the open arms versus the control (18.5 ± 3.4%; Figure 9c,d), indicating that HI increased anxiety-related behavior in these animals. Interestingly, in both cases, GH treatment induced an increase in the number of entries (3.1 ± 0.6, *p* < 0.05) and time spent in the open arms (13.3 ± 2.6%, *p* < 0.05) when compared to the HI group.

In the rotarod test, at P62 (tertiary phase), the HI group exhibited impaired coordination (*p* < 0.01), as evidenced by a shorter latency to fall (57.2 ± 6.3 s) compared to the vehicle group (85.0 ± 5.5 s). In contrast, GH treatment effectively reversed this deficit (88.0 ± 7.6 s), Figure 9g.

## 3. Discussion

This study provides compelling evidence that GH exerts significant neuroprotective and immunomodulatory effects in the developing cerebellum following neonatal global hypoxia. Neonatal HI triggered a robust and sustained neuroinflammatory response in the developing cerebellum, persisting for at least seven days post-insult, as reflected by the temporal dynamics in the expression of proinflammatory mediators. This prolonged immune activation was associated with persistent reactive gliosis, Purkinje cell loss, and myelin disruption in adulthood, and the resulting harm provoked a faulty execution in the behavioral tests in comparison to the controls. In turn, GH exerted a biphasic modulation of inflammation—transiently proinflammatory during the initial latent phase (hours), which then shifted towards anti-inflammatory effects in the secondary phase (days). Ultimately, GH significantly attenuated neuroinflammatory markers and glial reactivity, while promoted pro-survival signaling and improved long-term functional outcomes. All these findings underscore the therapeutic potential of GH in ameliorating cerebellar dysfunction and long-term sequelae associated with perinatal asphyxia.

The cerebellum is critically involved in the fine-tuning of motor functions, including coordination, postural control, and balance. Cerebellar injury leads to ataxia, characterized by impaired motor coordination [31], largely attributable to the degeneration of Purkinje neurons, which are among the most selectively vulnerable neuronal populations to HI insult [32]. Furthermore, cerebellar damage sustained during the neonatal period or in preterm infants has been implicated in long-term neurodevelopmental deficits, including cognitive and behavioral dysfunctions [6].

Hypoxic–ischemic injury to the cerebellum in neonatal rats elicits a robust neuroinflammatory response, typified by pronounced microglial activation and upregulated expression of phosphorylated NF-κB (pNF-κB), TLR4, TNF-α, IL-1β, iNOS, and their corresponding receptors TNF-R1 and IL-1R1, which are specifically localized to Purkinje neurons. These pro-inflammatory mediators remain elevated for approximately seven days following the HI insult, indicating a sustained neuroinflammatory response within the cerebellum [20,21]. This persistent inflammatory environment contributes to the degeneration of developing Purkinje neurons and a significant reduction in the thickness of both the molecular and granular layers of the cerebellar cortex [20,21,32]. In line with these findings, our previous study using a chicken embryo model demonstrated that HI exposure leads to pronounced alterations in cerebellar cytoarchitecture, including changes in layer size and organization [13].

To elucidate the molecular mechanisms underlying cerebellar inflammation, Yao et al. (2013) [20] demonstrated that TLR4 signaling plays a central role in hypoxia-induced microglial activation. This process is regulated by HIF-1α, which promotes the release of pro-inflammatory mediators through activation of the NF-κB signaling pathway. NF-κB is a key transcriptional regulator orchestrating the expression of numerous pro-inflammatory cytokine genes [33].

In accordance with previous findings [5], our results demonstrated a significant upregulation of HIF-1α, TLR4, IL-1β, and TNF-α, along with the activation of NF-κB, TNF-R1, and TNF-R2 signaling pathways in the cerebellum of neonatal rats following HI insult. TNF-α exerts pleiotropic effects through its receptors, TNF-R1 and TNF-R2, both of which modulate critical cellular processes, including survival, proliferation, immune activation, and inflammation [5]. Under physiological conditions, TNF-α contributes to neuroplasticity and myelination; however, its pathological overexpression has been implicated in excitotoxicity, chronic neuroinflammation, and disruption of BBB integrity [33].

Notably, the expression of IL-1β, TNF-α, and TLR4 increased rapidly upon hypoxia, whereas the upregulation of IL-6, COX-2, and iNOS was delayed, beginning approximately three hours after reoxygenation in the latent phase. This temporal distinction may be attributed to the regulatory mechanisms governing IL-6 expression, which is known to be modulated by transforming growth factor-beta (TGF-β) in synergy with IL-1β and TNF-α during inflammatory processes [34].

In our model, the temporal profile of the inflammatory response followed a bell-shaped curve, reflecting a marked and sustained upregulation of IL-1β, TNF-α, COX-2, and IL-6, alongside the activation of NF-κB and TNF-R1 signaling pathways. throughout the early and late secondary phases of injury, lasting several days. In contrast, iNOS displayed a distinct peak-like expression pattern, rather than a gradual curve, consistent with previous reports indicating that iNOS upregulation begins as early as 4 h post-insult, peaks between 12 and 48 h, and typically declines by day 7. These dynamics appear to vary depending on the affected brain region and the nature of the HI insult [35]. Furthermore, markers of microglial/macrophage polarization—CD86 (M1, pro-inflammatory) and CD206 (M2, anti-inflammatory)—were concurrently elevated, suggesting the presence of a mixed activation phenotype during the cerebellar inflammatory response in the neonatal brain [36].

To trace the impact of GH treatment on the cerebellar inflammatory response following HI, we identified a biphasic modulatory effect, as mentioned above. During the latent phase, GH treatment further enhanced the expression of pro-inflammatory mediators IL-1β, IL-6, TLR4, and iNOS beyond levels observed with HI alone, while TNF-α and COX-2 expression remained elevated. Additionally, GH activated key intracellular signaling cascades, including Akt, Erk1/2, NF-κB, TNFR1, and TNFR2. Notably, GH increased expression of CD86 while reduced that of CD206, indicating a shift toward a pro-inflammatory microglial/macrophage phenotype. These findings suggest that GH exerts an early immunostimulatory role, potentially functioning as a priming factor during the acute immune response [25,37]. Previous studies have implicated GH in the regulation of macrophage activation, enhancing innate immune functions such as reactive oxygen species production, proteolytic activity, and mitochondrial oxidative phosphorylation [38]. Further investigations are needed to elucidate the mechanisms through which GH modulates acute neuroinflammation and microglial activation in the HI neonatal brain.

As GH treatment advanced during the early and late secondary phases, it provoked a significant reduction in the expression of key inflammatory mediators, including IL-1β, TNF-α, IL-6, TLR4, COX-2, and iNOS, which ultimately promoted a microglial/macrophage polarization shift towards the anti-inflammatory CD206 phenotype at the end of the treatment. Moreover, GH treatment robustly activated the Akt signaling pathway throughout the treatment period, highlighting its central role among the primary neuroprotective mechanisms elicited by GH [39,40]. GH also modulated the activation of NF-κB and Erk1/2 signaling cascades. We propose that the pronounced activation of Akt results not only from direct GH receptor engagement but also indirectly through the induction of other receptors, such as TNF-R2, or growth factors like IGF-1, which concurrently activate the Akt pathway. These molecular events contribute to survival signaling, including upregulation of anti-apoptotic proteins such as Bcl-2, a well-established mediator of GH neuroprotective effects. Collectively, these findings indicate that GH promotes neuronal survival in neonatal HI through multifaceted modulation of inflammatory and pro-survival pathways.

Similar modulatory effects have been documented in other experimental neural lesions, such as the spinal cord injury model, where GH treatment attenuated the upregulation of TNF-α and other pro-inflammatory cytokines, thereby mitigating inflammation and facilitating sensory recovery [25]. These findings are consistent with observations in the cerebellum of chicken embryos, in which GH enhanced the expression of trophic factors such as IGF-1, VEGF, and BDNF, concurrently reducing pro-inflammatory cytokine levels, including TNF-α, and promoting the survival of both Purkinje and granule neurons [13]. Within the cerebellum, it is well established that TNF-α modulates the expression of IGF-1 and several components of the IGF-1 system, thereby influencing cerebellar cytoarchitecture by affecting granule cell migration [41]. Moreover, TNF-α plays a pivotal role in modulating the neural activity of Purkinje cells, enhancing their excitability [42]. Given the integral role of TNF-α in orchestrating inflammatory responses and neuronal function, our data support the notion that GH treatment may have a therapeutic potential to mitigate TNF-α and IL-1β-mediated damage within the CNS, particularly in the vulnerable developing cerebellum.

Considering the endogenous response of GH and IGF-1 against HI, Nieto-Sampedro et al. (1982) [43] demonstrated that the brain releases endogenous neurotrophins between 3 and 10 days after traumatic brain injury, with this increase being significantly greater in immature rats compared to adults. Both GH and IGF-1 are key trophic factors that play essential roles during CNS development [44,45]. In our study, cerebellar expression levels of GH, GHR, IGF-1, and IGF-1R were robustly upregulated in response to HI, supporting our previous findings that locally expressed GH and IGF-1 increase following HI and is crucial for cell survival as an intrinsic neuroprotective mechanism [13,39,46]. Moreover, we also reported that GH partially regulates IGF-1 gene expression and plays a critical role in mediating GH protective effects on cerebellar granule cells after HI, evidenced by a significant increase in cell mortality after GH was knocked-down in chicken cerebellar cultures [46]. In the present study, IGF-1 proved to be a crucial mediator in response to GH treatment, highlighting the need for further investigation to differentiate between the neuroprotective direct effects of GH from those mediated by IGF-1.

Our findings indicated that under control (normoxic) conditions in the neonatal rat, subcutaneously injected GH rapidly distributed throughout the body and liver and was capable of crossing the BBB to reach cerebellar tissue within a short timeframe [13]. GH administration influenced the gene expression of its receptor in neonatal rat liver, aligning with developmental studies that demonstrate coordinated expression of IGF-1 and GHR genes, which underlies tissue-specific IGF-1 expression [44]. The GH/IGF-1 axis plays a critical role during brain development, promoting neural precursor proliferation, neurogenesis, and glial differentiation through paracrine mechanisms [47]. In the neonatal cerebellum, GHR signaling activates downstream pathways including STAT5, Erk1/2, and PI3K/Akt, consistent with prior studies showing GH-induced phosphorylation of STAT5 in rodent brains [48,49]. Notably, STAT5 activation localizes predominantly to Purkinje and granule layers, without detectable signal in the molecular layer [49]. Our previous work in chicken embryos exposed to HI further confirmed that GH was able to cross the BBB and colocalized with GHR in cerebellar layers and deep nuclei, highlighting their involvement in neuroprotective and reparative roles [13].

Activation of STAT5 is particularly significant as this transcription factor mediates IGF-1 production [50]. Our results corroborated that GH-induced STAT5 activation in the neonatal cerebellum was accompanied by increased local IGF-1 synthesis. IGF-1 is widely expressed across brain cell types during the perinatal period, exerting essential roles in Purkinje neuron survival and granule cell precursor differentiation [47,51]. Disruption of IGF-1 receptor signaling leads to cerebellar hypotrophy, hypoplasia, and hypofoliation, which impairs motor functions [51]. Despite these critical roles, the consequences of GHR signaling inhibition during brain development remain insufficiently explored. Evidence suggests that impaired GHR signaling may alter stress responses, growth patterns, and cognitive function in adulthood [45]. Early studies demonstrated that bGH administration corrected molecular and enzymatic alterations in the neonatal rat cerebellum and cortex after thyroidectomy, suggesting a developmental role for GH in the brain [52]. Collectively, these findings emphasize the effects of GH upon the local GH/IGF-1 axis in the cerebellum during early development and the importance of intact GHR signaling for proper cerebellar maturation and function.

In our study, neonatal HI elicited pronounced and persistent microglial activation within the cerebellum, which remained evident in adulthood. This response was reflected by an increased density of Iba-1^+^ cells and morphological alterations consistent with activation, including soma hypertrophy and enhanced process complexity—features that constitute well-established hallmarks of microglial/macrophage reactivity [36]. In contrast, GH treatment markedly reduced the number of Iba-1^+^ cells and attenuated their hypertrophic morphology in the chronically injured cerebellum. While this analysis offers meaningful information on microglial reactivity, it remains limited in distinguishing between polarization phenotypes or regional variability within the cerebellum. Together with the observed expression downregulation of pro-inflammatory factors during the neonatal period, these findings support a modulatory role of GH on microglial proliferation and activation in response to HI [25,37]. Given the essential role of microglia in the pruning and maturation of Purkinje neurons via orchestrated programmed cell death and phagocytosis [22], we propose that GH capacity to regulate microglial activation contributes to preserving Purkinje cell integrity.

In this study, neonatal HI elicited pronounced microglial activation, which was still present in the adult cerebellum, evidenced by an increased density of Iba-1-positive cells and morphological changes consistent with activation, including hypertrophy and enhanced process complexity. These features are established hallmarks of microglia/macrophage activation [36]. In clear contrast, GH treatment significantly reduced microglial cell numbers and attenuated microglial hypertrophy in the chronically injured cerebellum. Coupled with the observed downregulation of pro-inflammatory factors expression during the neonatal period, these data support a modulatory role of GH on microglial proliferation and activation in response to HI [25,37].

GFAP is a well-established marker of astrogliosis following CNS injuries such as HI [53]. Our study demonstrated that neonatal HI induced significant morphological changes in adult astrocytes, including Bergmann glia, evidenced by increased GFAP immunoreactivity in the molecular and granular layers, as well as in the cerebellar white matter, compared to controls and GH-treated groups. These findings align with previous reports describing elevated GFAP expression and altered astrocytic morphology in hypoxia-damaged cerebellum [11,32,54]. Notably, GH treatment significantly mitigated astrogliosis by reducing GFAP immunoreactivity and normalizing astrocyte morphology across these regions, including the cerebellar white matter [25,37].

It has been reported that HI to the forebrain causes cerebellar myelination defects as well as damage to granule and Purkinje cells, with microglia and astrocytes contributing to pathogenesis in the rat [11,32,55]. White matter is especially vulnerable due to death of immature oligodendrocytes prior to myelination onset and subsequent axonal degeneration [55,56]. Consistent with these findings, we observed a significant reduction in MBP immunoreactivity in the adult cerebellum of rats subjected to neonatal HI. While most studies have focused on white matter, our analysis aimed particularly in the molecular and granular layers—rich in dendrites and axons of Purkinje and granule neurons—showing that HI predominantly disrupted myelination in these fibers, as mossy and climbing inputs appeared demyelinated upon entering the cortex [55]. Although MBP in white matter was not quantified, a visible reduction was also evident, suggesting widespread myelination deficits. On the other hand, GH treatment markedly improved MBP immunoreactivity across all regions, including white matter, indicating a neuroprotective effect (likely mediated by IGF-1), which promoted oligodendrocyte proliferation and myelin synthesis [57]. Preserving axonal myelination is essential to maintain cerebellar structure and function, particularly in view of the integrative role of Purkinje neurons in cerebellar output.

Calbindin is a well-established marker of Purkinje neurons and plays a crucial role in neuronal survival and calcium homeostasis [11]. Here, we found that the HI group exhibited a pronounced disorganization of Purkinje cell dendritic arborization and increased neuronal loss—features absent in the control and GH-treated groups—suggesting disrupted neuronal maturation and impaired connectivity as consequences of neonatal HI. Conversely, our results demonstrated that GH treatment enhanced Purkinje cell survival, as evidenced by increased calbindin immunoreactivity in the adult cerebellum. These observations are in line with previous reports, in the chicken embryo model, that GH administration restored the width of the external granular layer and molecular layer, and improved the survival of both Purkinje and granule neurons following HI [13].

The effect of HI upon neuronal microtubule structure was examined, and IHC results revealed a reduction in β-III-tubulin immunoreactivity in the granular layer, where dendrites of granule neurons and axons of Purkinje cells are located. In addition, pronounced disorganization of Purkinje cell dendritic arborization was evident, with increased β-III-tubulin immunoreactivity in the molecular layer and reduced β-III-tubulin presence in the white matter, further indicating cytoskeletal disruption following HI. This suggests that HI may impair dendritic orientation, synaptic integrity, and intracellular transport—processes highly dependent on intact microtubule networks [58,59]. In this context, the increased β-III-tubulin immunoreactivity may result from microglial dysfunction, which is critical for dendritic pruning and Purkinje cell layer refinement during postnatal development [22]. Conspicuously, β-III-tubulin staining also revealed ectopic somas of Purkinje neurons, associated with an abnormal localization, indicating that hypoxia injury disrupted their migratory trajectory toward the cerebellar cortex. This observation is consistent with previous reports in mutant mice exhibiting cerebellar malformations, such as *reeler* mice [60], as well as in the HI chicken cerebellum [13]. Our data showed that GH treatment counteracted hypoxia-induced alterations in β-III-tubulin expression across all cerebellar regions examined, promoting dendritic and axonal growth in both Purkinje and granule neurons. These findings support the notion that GH exerts anti-inflammatory and neuroprotective effects during the neonatal period, thereby preserving cerebellar cytoarchitecture and preventing long-term structural disruption of key neuronal populations.

The cerebellum has been described as particularly vulnerable to hypoxic and ischemic insults [32,55]. Moreover, cerebellar injury in preterm infants or during the neonatal period has been associated with long-term cognitive and behavioral dysfunction [6]. In the present study, we observed that HI at postnatal day 2 results in persistent motor coordination deficits, which were assessed up to postnatal day 62. This observation aligns with the findings of Leroux et al. (2022) [55], who employed a murine model of apnea of prematurity involving repeated episodes of intermittent hypoxia from the same postnatal stage (P2), and demonstrated that, despite partial structural compensation within the cerebellum, significant long-term functional impairments remain, including deficits in motor coordination and spatial learning. Although compensatory mechanisms are activated to preserve cortical layering, persistent morphological and functional abnormalities—particularly within Purkinje cells—indicate that early-life HI insults induce lasting disruptions in cerebellar maturation [55]. These alterations, such as dendritic densification, hypomyelination, and aberrant afferent innervation, likely contribute to sustained motor and cognitive deficits [55]. Importantly, such cerebellar damage results in long-term consequences that are not fully resolved by delayed anatomical recovery, as persistent abnormalities in Purkinje cell morphology and connectivity disrupt cerebellar output and contribute to behavioral deficits [55]. To our knowledge, this study is the first to demonstrate that a five-day systemic administration of GH significantly mitigates the molecular, structural, and functional sequelae of neonatal HI, leading to improved motor coordination comparable to control animals. These findings underscore the therapeutic potential of GH in promoting cerebellar repair and functional recovery following perinatal hypoxia, likely through modulation of neurodevelopmental processes critical for motor function restoration.

Our data indicate that GH treatment attenuates neuroinflammation and gliosis, not only improving motor coordination but also reducing anxiety-like behaviors. Notably, GH deficiency has been associated with increased anxiety and impaired fear memory in the context of post-traumatic stress disorder, consistent with GHR expression in the amygdala [61]. These results align with previous reports demonstrating the neurorestorative effects of GH in models of experimental traumatic brain injury [24], spinal cord injury [25], and sciatic nerve transection [26], supporting a broad therapeutic potential across diverse forms of neural damage. In the present study, the observed behavioral improvements are likely mediated by the protective actions of GH on microglial and astrocytic activation, preservation of myelin integrity, and enhanced survival of Purkinje neurons in the adult cerebellum. Given the central role of Purkinje cells as primary integrators of cerebellar output, their preservation appears critical for maintaining cerebellar function and ensuring favorable motor and cognitive outcomes.

Our findings highlight the essential role of GH/IGF-1 signaling in neonatal hypoxia and cerebellar development, supporting GH as a potential therapeutic intervention. Studies in transgenic spinocerebellar ataxia type 3 mice have demonstrated that IGF-1 therapy is less effective than GH treatment [62], likely reflecting distinct regulatory mechanisms by which GH and IGF-1 confer neuroprotection. Compared with other clinically evaluated neuroprotective strategies for neonatal hypoxia, such as therapeutic hypothermia [63], erythropoietin [64], IGF-1 [62], and melatonin [65], GH exhibits both overlapping and unique effects. While sharing anti-apoptotic, anti-inflammatory, antioxidant, and angiogenic properties, GH additionally activates the GH/IGF-1 axis and modulates the expression of a broad range of neurotrophic factors (IGF-1, IGFBP-2, BDNF, EPO, VEGF) across multiple brain regions, including the hippocampus, prefrontal cortex, and cerebellum [13,64,66]. This positions GH as both a potential alternative and an adjuvant therapy in neonatal hypoxic brain injury. Such a multifaceted neurotrophic profile is particularly relevant given the expanding recognition of cerebellar contributions to non-motor functions, mediated through its complex connectivity with the hypothalamus, thalamus, neocortex, and hippocampus [67].

Although the inclusion of an additional control group (Normoxia + GH, [24,25,26]) could further help to discern if GH administration has any effect upon several markers under normal conditions, we believe the results of this study clearly demonstrated the neuroprotective effects of GH after hypoxic injury. While it has been shown that GH exerts moderate modulatory actions under normoxia—such as the upregulation of angiogenic mediators like EPO that stabilize the BBB [64]—its protective effects are markedly enhanced during hypoxia, as demonstrated in this and previous studies [64]. On the other hand, despite the beneficial actions of GH treatment in the CNS, some concern still remains about the potential risk of promoting malignant tumors, diabetes, or angiogenesis-related diseases, which underscores the need for further studies to establish its dosing and long-term safety [68].

In conclusion, this study demonstrates that GH exerts potent neuroprotective and immunomodulatory effects in the developing cerebellum following neonatal global hypoxia. GH modulates the inflammatory response in a biphasic manner, initially promoting acute proinflammatory activity, then shifting to anti-inflammatory effects during the subacute phase. This regulation contributes to the attenuation of sustained neuroinflammation, gliosis, Purkinje cell loss, and myelin disruption observed in adulthood. Moreover, GH enhances pro-survival signaling pathways and improves long-term functional outcomes, including motor coordination and anxiety-like behavioral parameters. These findings support the therapeutic potential of GH for mitigating cerebellar dysfunction and long-term neurological deficits resulting from perinatal hypoxia injury.

## 4. Materials and Methods

### 4.1. Animals

*Wistar* rats at postnatal day 2 (P2) were used, with equal numbers of males and females randomly included in all experimental groups. Pups remained with their dam under controlled conditions (12-h light/dark cycle, 20–22 °C, 50–60% humidity) with food and water ad libitum. All procedures were approved by the Institute of Neurobiology’s Research Ethics Committee (protocol #141-A) and complied with NOM-062-ZOO-1999/SAGARPA. Rats were randomly assigned to two experimental cohorts: (1) normoxia (Nx) and hypoxia–ischemia (HI) groups, and (2) normoxia + vehicle (Veh), hypoxia-reoxygenation + vehicle (H-Ox-Veh), and hypoxia-reoxygenation + GH (H-Ox-GH) groups. To minimize litter effects, four pregnant females were used as independent randomization units, and pups from each litter were allocated across the different experimental groups.

### 4.2. Experimental Design

**Figure 10:**
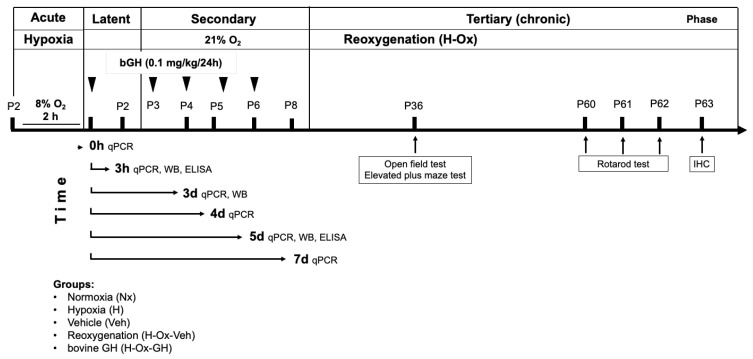
Experimental design to assess the effect of neonatal hypoxia and GH treatment. One cohort of experimental groups of rats was subjected to gene expression analysis (qPCR) and protein analysis (WB and ELISA) to evaluate the extent of the inflammatory response and growth factor expression in the cerebellum. Analyses were conducted at Time = 0 h (acute phase, P2), two hours post-hypoxia, and at Time = 3 h (latent phase, P2), three hours post-injury, following reoxygenation (H-Ox). Further evaluations were performed during the secondary phase at Time = 3 d (early secondary phase, P4), Time = 4 d (secondary phase, P5), Time = 5 d (late secondary phase, P6), and Time = 7 d (late secondary phase, P8), following hypoxia and bovine growth hormone treatment (bGH, 0.1 mg/kg/24 h). A second cohort of animals was used for behavioral testing during the tertiary (chronic) phase at P36, using the open field and the elevated plus maze tests. Additionally, the rotarod test was performed at P60–62, and finally immunohistochemical analyses were conducted at P63. Arrows indicate the temporal sequence of experimental phases and procedures. Abbreviations: P, postnatal day; qPCR, quantitative polymerase chain reaction; WB, Western blot; ELISA, enzyme-linked immunosorbent assay; IHC, immunohistochemistry.

Rats at P2 were exposed for 2 h to whole-body (global) hypoxia inside an acrylic chamber (Napco E Series, Model 302; LabRepCo, Horsham, PA, USA). The chamber was flushed with a gas mixture of 5% CO_2_ and 95% N_2_ until reaching a final oxygen concentration of 8%, continuously monitored with an O_2_ sensor (BioSpherix, Model ProOx 110; BioSpherix, Parish, NY, USA), and maintained at 37 °C, as previously described [21]. Afterwards, they recovered under normoxia (21% O_2_, H-Ox) during GH treatment and until the experiment ended. Control groups remained under normoxia and received saline solution (Veh) as treatment (Figure 10).

GH treatment effects were analyzed across several phases of hypoxia [16]—acute (HI), latent, secondary, and tertiary (chronic)—by measuring inflammatory markers and growth factors through quantitative polymerase chain reaction (qPCR, Table 1), Western blotting (WB, Table 2), and enzyme-linked immunosorbent assay (ELISA, Figure 10). Results are presented by phases as follow: P2, 2 h post-hypoxia (Time = 0 h, acute phase, P2), then at 3 h post-injury (Time = 3 h, latent phase, P2), and subsequently during the secondary phase at P4 (Time = 3 d, early secondary phase), P5 (Time = 4 d, secondary phase), P6 (Time = 5 d, late secondary phase), and P8 (Time = 7 d, late secondary phase), as shown in Figure 10.

Behavioral studies were conducted during the tertiary phase (P36–P63), specifically at P36 (open-field and elevated plus maze), and P60–62 (rotarod test), followed by immunohistochemistry at P63 (Figure 10).

Bovine GH (bGH; 0.1 mg/kg; NHPP AFP10325C, Harbor-UCLA Medical Center, Torrance, CA, USA) was administered subcutaneously (s.c.) every 24 h, starting immediately after HI, for five consecutive days until P6. The selected dose (0.1 mg/kg/day for 5 days) was based on previous reports demonstrating the neuroprotective effects of GH in both neonatal and adult models of brain injury [13,24]. Animals were then euthanized at specific time points for tissue collection and analysis (Figure 10). In the latent phase, samples were taken at Time = 3 h (latent phase, P2) after a single bGH dose. During the secondary phase, tissues were collected at Time = 3 d (early secondary phase, P4; 3 h after the third dose), Time = 4 d (secondary phase, P5; 24 h after the third dose), Time = 5 d (late secondary phase, P6; 3 h after the fifth dose), and Time = 7 d (late secondary phase, P8; 48 h after the fifth dose), Figure 10.

### 4.3. Determination of Bovine GH and Rat IGF-1 Concentrations by ELISA

bGH concentrations (ng/mL) were quantified using a bGH ELISA kit (MyBiosource, Inc., San Diego, CA, USA, cat. no MBS2086954) in serum (20 µL) obtained by cardiac puncture from P2 rats at 0, 30, 60, 120, or 180 min after receiving a single subcutaneous dose (0.1 mg/kg) of bGH. Animals were euthanized at those times, brain tissues were collected, and protein content was determined by the Bradford method (Bio-Rad). Cerebellar IGF-1 was quantified in extracts (5 µg of total protein) with a commercial ELISA kit (Thermo Scientific, Waltham, MA, USA, cat. no. ERIGF1). In addition, IGF-1 levels were also measured at P6, after HI and five bGH doses (Time = 5 d, Figure 10). Results were expressed as ng/mg of tissue protein.

### 4.4. RNA Extraction and RT-qPCR

Total RNA was extracted from cerebellum using TRIzol Reagent (Invitrogen, Waltham, MA, USA, cat. no. 15596018), then purified using Direct-zol RNA Mini Prep kit (Zymo Research Corp., Irvine, CA, USA, cat. no. R2072) and treated with DNase on-column, following the manufacturers’ instructions. For each sample, cDNA was synthesized using 1 µg of total RNA and 200 U of M-MLV Reverse Transcriptase (Invitrogen, Waltham, MA, USA, cat no. 28025013), at 37 °C for 60 min.

Real-time qPCR (RT-qPCR) was conducted using a QuantStudio System (Applied Biosystems, Foster City, CA, USA; QuantStudio version 3.0) with Maxima SYBR-Green Master Mix reagent (Maxima; Thermo Fisher Scientific, Waltham, MA, USA, cat. no. K0252) in a 10 µL final volume containing 3 µL cDNA (diluted 1:10 for hypoxanthine phosphoribosyltransferase [HPRT] and 1:3 for other genes) and 0.5 µL (0.5 µM) of each specific primer (forward and reverse).

Primers were designed to span exon-exon boundaries where possible (Table 1) using the BLAST primer algorithm (NCBI Primer-BLAST, version 2.13.0). Efficiency was confirmed with standard curves in cDNA for cerebellum. Reactions were performed under the following conditions: initial denaturation at 95 °C for 10 min; followed by 40 cycles at 95 °C for 15 s, 60 °C for 30 s, and 72 °C for 30 s. Dissociation curves were included after each qPCR experiment to ensure primer specificity. The relative abundance of the studied mRNAs was calculated using the comparative threshold cycle method and 2^−∆∆CT^ formula [69]. Quantification was expressed relative to HPRT mRNA [70].

### 4.5. SDS-PAGE and Western Blot Analysis (WB)

Cerebellar tissues were homogenized in RIPA lysis buffer (pH 7.5; Abcam, Cambridge, UK, ab156034) containing a protease and phosphatase inhibitor cocktail (Sigma-Aldrich, Darmstadt, Germany, cat. no MSSAFE-1VL), using an ultrasonicator (GE 130PB, Cole-Parmer, Vernon Hills, IL, USA) at a power amplitude of 6 for 20 s. Supernatants were collected after centrifugation (12,500× *g* for 15 min), and total proteins determined with the Bradford assay (Bio-Rad). Samples (35 µg/lane) were analyzed in 10.0% SDS-PAGE gels under reducing conditions and transferred onto nitrocellulose membranes (Bio-Rad) at a constant current of 200 mA for 1.5 h. Membranes were blocked with 5% non-fat dry milk (Bio-Rad, Hercules, CA, USA, cat. no. 170-6404) in Tris-buffered saline (TBS, 0.05 M Tris; 0.15 M NaCl, pH 7.6) for 1 h at room temperature (RT), and then incubated overnight at 4 °C with the corresponding primary antibodies (Table 2) diluted in TTBS (1 × TBS with 0.05% Tween [vol/vol]). After washing with TTBS, the membranes were incubated with the respective horseradish peroxidase (HRP)-conjugated secondary antibodies (Table 2), for 2 h a RT. Immunoreactive (IR) bands were visualized using an ECL blotting detection reagent (Amersham-Pharmacia, Buckinghamshire, UK) on autoradiography film (Fujifilm, Tokyo, Japan). Kaleidoscope molecular weight markers (Bio-Rad, Hercules, CA, USA, cat. no. 1610375) were used for molecular mass determination. Images were captured on a Gel Doc EZ Imager (Bio-Rad, Hercules, CA, USA), and optical densities from immunoreactive bands were analyzed with Image Lab software (Bio-Rad, Hercules, CA, USA, version 6.1.0). Band immunoreactivities (IR) were normalized to the corresponding glyceraldehyde 3-phosphate dehydrogenase (GAPDH), and the results were expressed as percentage of optical density (% O.D.) relative to control values.

### 4.6. Behavioral Analyses

Experimental groups (eight animals in each) were submitted to a battery of behavioral tests (as described below) under controlled conditions: RT (20–22 °C), humidity (50–60%), and light intensity (dim illumination). To prevent circadian variations, assessments were always performed during the rat active (dark) phase at the Behavioral Analysis core facility. Animals were housed in the examination room one week before experiments and habituated for at least 1 h prior to each test.

#### 4.6.1. Open Field Test

Locomotor activity was assessed at P36 using the SuperFlex/Fusion system (Digiscan Animal Activity Monitors, Omnitech Electronics, Columbus, OH, USA) in a sound-isolated room. Each rat was placed individually in a well-lit Plexiglas chamber (40 × 40 × 30 cm) for 5 min and allowed to explore freely. Movements were recorded by 16-photocell SuperFlex sensors (three paired arrays) and transmitted to the SuperFlex Node. Data were analyzed with Fusion Software (v5.3). Total distance (m), as well as horizontal and vertical activity counts were analyzed to evaluate locomotor performance [71].

#### 4.6.2. Elevated Plus Maze Test

Anxiety-like behavior was evaluated at P36 using the elevated plus maze, consisting of four arms (50 × 10 cm) arranged in a cross around a central platform (10 × 10 cm) elevated 50 cm above the floor. Two arms were enclosed by 40-cm-high walls; the others were open. Illumination was ~100 lx in open arms and ~35 lx in closed arms. Each rat was placed at the maze center, facing an open arm, and recorded for 5 min. The maze was cleaned between sessions. Behavior was analyzed with SMART 3.0 software (Panlab Harvard Apparatus, Barcelona, Spain), measuring: (1) number of entries into open arms and (2) time spent in open arms. To count as an entry required all four paws crossed into an arm [71].

#### 4.6.3. Rotarod Test

Motor coordination and balance were evaluated using a rotarod apparatus (IITC Inc. Life Science, Woodland Hills, CA, USA). Testing consisted of four trials/day for three consecutive days (P60—P62). The rod accelerated constantly and uniformly from 5 to 65 rpm in 180 s. The apparatus was thoroughly cleaned after the removal of each animal. Data analysis was based on the maximal time (latency to fall) that the rat was able to stay on the rotating rod [71].

### 4.7. Immunohistochemistry (IHC) and Confocal Microscopy

Adult rats (P63) were euthanized with a pentobarbital overdose (150 mg/kg, PiSA, Guadalajara, Mexico, cat. no. 4003178) and trans-cardially perfused with saline solution and 4% paraformaldehyde in PBS. Cerebella were postfixed overnight and cryoprotected in sucrose-PBS at 4 °C. Parasagittal sections (15 μm) were cut on a freezing cryostat (Leica CM 1850, Biosystems, Wetzlar, Germany) and mounted on (3-aminopropyl) trimethoxysilane-coated slides (Sigma-Aldrich, Darmstadt, Germany, cat. no. 440140).

Sections were incubated overnight at 4 °C with primary antibodies (Table 2) diluted in 1% non-fat dry milk in 0.05% Tris phosphate-buffered saline (TPBS), followed by secondary antibodies (1% milk in TPBS) for 3 hat RT. Negative controls without primary antibodies were included. Nuclei were labeled with To-Pro3 (1:1000, Invitrogen, Waltham, MA, USA cat. no. T3605), and sections were mounted with Fluoro-Gel (Laborimpex, Brussels, Belgium, cat. no. 17985-11). Z-stack images were captured using a Zeiss LSM 780 DUO confocal microscope (Carl Zeiss AG, Oberkochen, Germany).

Immunofluorescence analysis encompassed the entire field of view at each magnification, including the molecular, Purkinje cell, and granular layers of the cerebellar cortex. Image quantification was performed using Fiji software (version 2.14.0, NIH, Bethesda, MD, USA) [72] on 3–4 slices per group, analyzing 3–5 representative fields per slice. GFAP and MBP were quantified at 10×, Calbindin at 25×, and Iba-1^+^ and βIII-tubulin at 40× magnification, according to marker type, either by mean fluorescence intensity or by cell counting. Integrated density units were calculated as the sum of pixel values (RawIntDen) to measure immunoreactivity (IR). Microglial morphology was further assessed at the single-cell level using fractal analysis and individual skeletal analysis [73].

### 4.8. Statistical Analysis

Data are presented as mean ± standard error of the mean (SEM). Normality and homogeneity of variances were evaluated using the Shapiro–Wilk and Levene’s tests, respectively. Parametric analyses included Student’s *t*-test for two-group comparisons and one-way or two-way ANOVA followed by Tukey’s or Dunnett’s post hoc tests. Nonparametric data were analyzed using the Kruskal–Wallis test. ANOVA was used for complete datasets, whereas a mixed-effects model (REML) was applied when missing values occurred (e.g., due to mortality or bioethical euthanasia) to enable valid comparisons without excluding entire subjects. Asterisks (*) indicate significant differences compared with control, determined by one-way or two-way ANOVA with Tukey’s post hoc test (* *p* < 0.05; ** *p* < 0.01; *** *p* < 0.001). At symbols (@) indicate significant differences within the reoxygenation group (H-Ox-Veh) across time points [Time = 0 h (acute phase, P2), Time = 3 h (latent phase, P2), Time = 3 d (early secondary phase, P4), Time = 4 d (secondary phase, P5), Time = 5 d (late secondary phase, P6), Time = 7 d (late secondary phase, P8)] (@ *p* < 0.05). Hashtags (#) indicate significant differences within the GH-treated group (H-Ox-GH) across the same time points (# *p* < 0.05). Ampersands (&) indicate significance from pairwise comparisons using Student’s *t*-test (^&^ *p* < 0.05; ^&&^ *p* < 0.01). These symbol-based temporal comparisons (@, #) were applied exclusively to mRNA expression analyses of inflammatory markers, GH, IGF-1, and their receptors (Figure 2 and Figure 3), whereas ampersands (&) were also used in other datasets to denote *t*-test pairwise comparisons.

Biochemical and gene expression analyses (qPCR, Western blotting, ELISA, and immunohistochemistry) were performed in two independent experiments, whereas behavioral evaluations (open field, elevated plus maze, and rotarod tests) were conducted in at least three independent experimental series. The sample size (*n* = 8 per group) was estimated using G*Power 3.1 (Heinrich Heine University, Düsseldorf, Germany, version 3.1.9.7) to achieve a statistical power of ≥80% (α = 0.05). The exact number of subjects per group and time point is specified in each figure, as it was adjusted since dropouts due to mortality or bioethical euthanasia have occurred. Outliers were identified and excluded using the ROUT method (Q = 1%) implemented in Prism Graph 10 (GraphPad Software, San Diego, CA, USA).

## Figures and Tables

**Table 1:** List of primers used for real-time PCR analysis.

Target Gene	Primer	Sequence	Size (bp)	Accession #
**Inflammatory Markers**				
IL-1β	Fwd	CACCTCTCAAGCAGAGCACAG	79	NM_031512.2
	Rev	GGGTTCCATGGTGAAGTCAAC		
TNF-α	Fwd	AAATGGGCTCCCTCTCATCAGTTC	111	NM_012675.3
	Rev	TCTGCTTGGTGGTTTGCTACGAC		
IL-6	Fwd	TCCTACCCCAACTTCCAATGCTC	79	NM_012589.2
	Rev	TTGGATGGTCTTGGTCCTTAGCC		
COX-2	Fwd	TGTATGCTACCATCTGGCTTCGG	94	NM_017232.4
	Rev	GTTTGGAACAGTCGCTCGTCATC		
iNOS	Fwd	CCTTGTTCAGCTACGCCTTC	179	NM_012611.3
	Rev	GGTATGCCCGAGTTCTTTCA		
TLR-4	Fwd	GTTGGATGGAAAAGCCTTGA	198	NM_019178.2
	Rev	CCTGTGAGGTCGTTGAGGTT		
**Growth factors**				
GH	Fwd	GGCCCAGCAGAGAACTGACAT	174	NM_001034848.2
	Rev	ATCAGAGCCTGGATGCCCTC		
GHR	Fwd	ATCTTTGGCGGGTGTTCTTA	78	NM_017094.2
	Rev	TAGCTGGTGTAGCCCCACTT		
IGF-1	Fwd	ACCTTGCAAAAGTGGTCCTG	163	NM_001082477.2
	Rev	AGGAATTTAGTGCAACCGAA		
IGF-1R	Fwd	GCCGTGCTGTGCCTGTCCTAAAAC	189	NM_001414181.1
	Rev	GCTACCGTGGTGTTCCTGCTTCG		
HIF-1 α	Fwd	TGCTTGGTGCTGATTTGTGAA	94	NM_024359.2
	Rev	TATCGAGGCTGTGTCGACTGAG		
HPRT	Fwd	GACCGGTTCTGTCATGTCG	61	NM_012583.2
	Rev	ACCTGGTTCATCATCACTAATCAC		

bp, base pair.

**Table 2:** Primary and secondary antibodies used for Western blot and Immunohistochemistry.

Target	Host/Type	Dilution	Source	Cat. No.
pSTAT5	Rabbit/polyclonal	1:1000 WB	Cell Signaling	9351S
pErk1/2	Mouse/monoclonal	1:1000 WB	Cell Signaling	9106S
pAkt	Rabbit/monoclonal	1:1000 WB	Cell Signaling	92715
Bcl-2	Mouse/monoclonal	1:1000 WB	Santa Cruz	Sc-509
pNFkB	Rabbit/monoclonal	1:1000 WB	Cell Signaling	3033S
CD86	Rabbit/polyclonal	1:1000 WB	Abcam	Ab112490
CD206	Rabbit/polyclonal	1:1000 WB	Abcam	Ab64693
TNF-R1	Goat/polyclonal	1:1000 WB	Santa Cruz	sc-1068
TNF-R2	Goat/polyclonal	1:1000 WB	Santa Cruz	Sc-1072
Iba-1	Goat/polyclonal	1:500 IHC	Abcam	Ab5076
GFAP	Mouse/monoclonal	1:500 IHC	Millipore	MAB360
MBP	Rabbit/polyclonal	1:500 IHC	Invitrogen	PA5-78397
Β-III-Tubulin	Mouse/monoclonal	1:500 IHC	Abcam	Ab78078
Calbindin	Rabbit/monoclonal	1:500 IHC	Cell Signaling	2173S
HIF-1 α	Rabbit/monoclonal	1:1000 WB	Abcam	Ab51608
GAPDH	Rabbit/monoclonal	1:2000 WB	Cell Signaling	21182
Alexa Fluor 488	Rabbit/polyclonal	1:1000 IHC	Invitrogen	A11078
Alexa Fluor 555	Donkey/polyclonal	1:1000 IHC	Invitrogen	A31572
Alexa Fluor 594	Goat/polyclonal	1:1000 IHC	Invitrogen	A11012
Goat anti-Mouse IgG (H + L) Cross-Adsorbed Secondary Antibody, HRP	Goat/polyclonal	1:4000 WB	Thermo Fisher Scientific	G-21040
Goat anti-Rabbit Ig (H + L) Secondary Antibody, HRP	Goat/polyclonal	1:4000 WB	Invitrogen	656120
Rabbit anti-goat Ig (H + L) Secondary Antibody, HRP	Rabbit/polyclonal	1:4000 WB	Invitrogen	81-1620

WB, Western blot; IHC, Immunohistochemistry.

## Data Availability

The original contributions presented in the study are included in the article/Supplementary Material, further inquiries can be directed to the corresponding authors.

## Supplementary Materials

The following supporting information can be downloaded at: https://www.mdpi.com/article/10.3390/ijms262110671/s1.

## Abbreviations

The following abbreviations are used in this manuscript:

CNS	Central Nervous System
GH	Growth Hormone
HI	Hypoxic Injury
IGF-1	Insulin-like Growth Factor 1
TLR-4	Toll-like Receptor 4
IL-1β	Interleukin-1 beta
TNF-α	Tumor Necrosis Factor alpha
IL-6	Interleukin-6
COX-2	Cyclooxygenase-2
iNOS	Inducible Nitric Oxide Synthase
pNF-κB	Phosphorylated Nuclear Factor kappa B
pStat5	Phosphorylated Signal Transducer and Activator of Transcription 5
pErk1/2	Phosphorylated Extracellular Signal-Regulated Kinases 1/2
pAkt	Phosphorylated Protein Kinase B (Akt)
Bcl-2	B-cell lymphoma 2
TNF-R2	Tumor Necrosis Factor Receptor 2
Iba-1	Ionized Calcium-Binding Adapter Molecule 1
GFAP	Glial Fibrillary Acidic Protein
MBP	Myelin Basic Protein
BBB	Blood–Brain Barrier
Ca^2+^	Calcium Ion
PA	Perinatal Asphyxia
LPS	Lipopolysaccharide
IL-1R1	Interleukin-1 Receptor Type 1
TNFR1	Tumor Necrosis Factor Receptor 1
UNICEF	United Nations International Children’s Emergency Fund
MO	Molecular Layer
GR	Granular Layer
WM	White Matter
RawIntDen	Integrated Density
bGH	Bovine Growth Hormone
P	Postnatal day
HIF-1α	Transcription Factor Hypoxia-Inducible Factor-1 alpha
s.c.	Subcutaneous
IR-	Immunoreactivity
TGF-β	Transforming Growth Factor-beta
IGF-2	Insulin-like Growth Factor 2
IGFBP-2	Insulin-like Growth Factor Binding Protein 2
EPO	Erythropoietin
VEGF	Vascular Endothelial Growth Factor
BDNF	Brain-Derived Neurotrophic Factor
ELISA	Enzyme-Linked Immunosorbent Assay
qPCR	Quantitative Polymerase Chain Reaction
WB	Western blotting
HPRT	Hypoxanthine Phosphoribosyltransferase
GAPDH	Glyceraldehyde 3-phosphate dehydrogenase
IHC	Immunohistochemistry
TBS	Tris-Buffered saline
