# Supplementary material for: Growth Hormone Effects on Hypoxia-Induced Neuroinflammation in the Developing Cerebellum
Source: Int J Mol Sci. 2025 Nov 1;26(21):10671. doi: 10.3390/ijms262110671 (PMC12610270; doi:10.3390/ijms262110671)
Supplement: Supplementary file 1 [file ijms-26-10671-s001.zip › ijms-3842587-supplementary.pdf]

Supplementary Material

Supplementary Figures

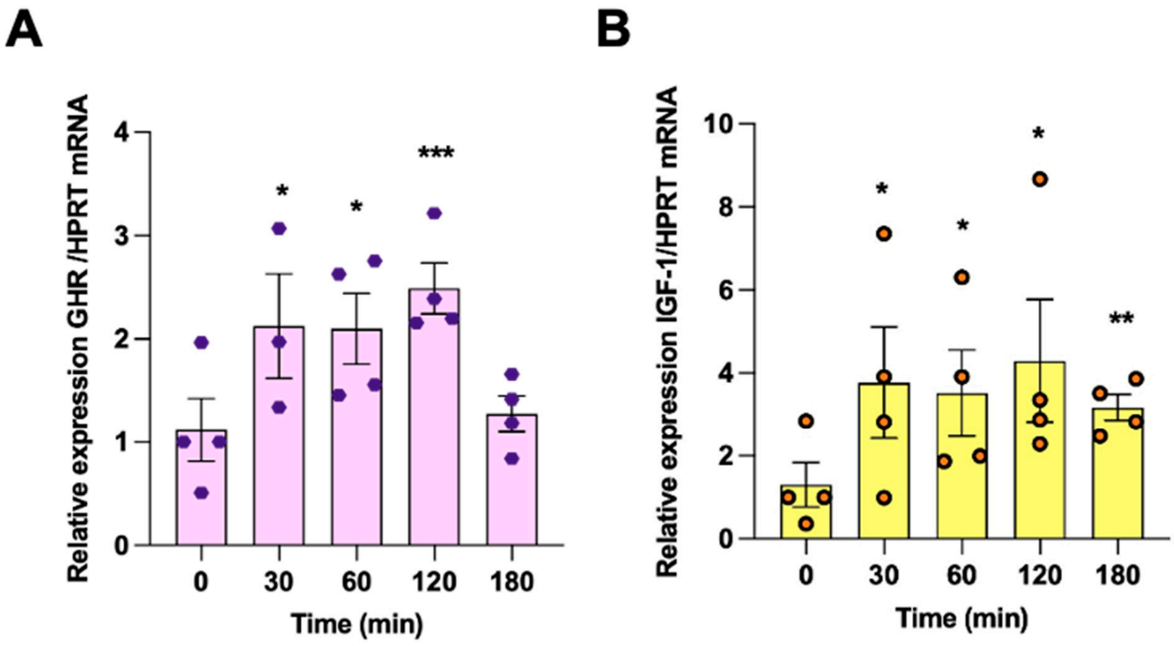

Supplementary Figure S1. Relative gene expression of GHR and IGF-1 in liver tissue of neonatal rats. Levels were measured in the liver at 0, 30, 60, 120, and 180 minutes after a subcutaneous injection of a dose of bGH (0.1 mg/kg) on postnatal day 2 (P2). (A) Relative change of GHR mRNA (n = 4/group). (B) Relative change of IGF-1 mRNA (n = 4/group). Hypoxanthine phosphoribosyl-transferase (HPRT) was used as the housekeeping gene. Results are shown as mean ± SEM. Asterisks indicate significant differences compared with control (0 min), determined by one-way ANOVA with Dunnett post hoc test (*$p < 0.05$; **$p < 0.01$; ***$p < 0.001$).

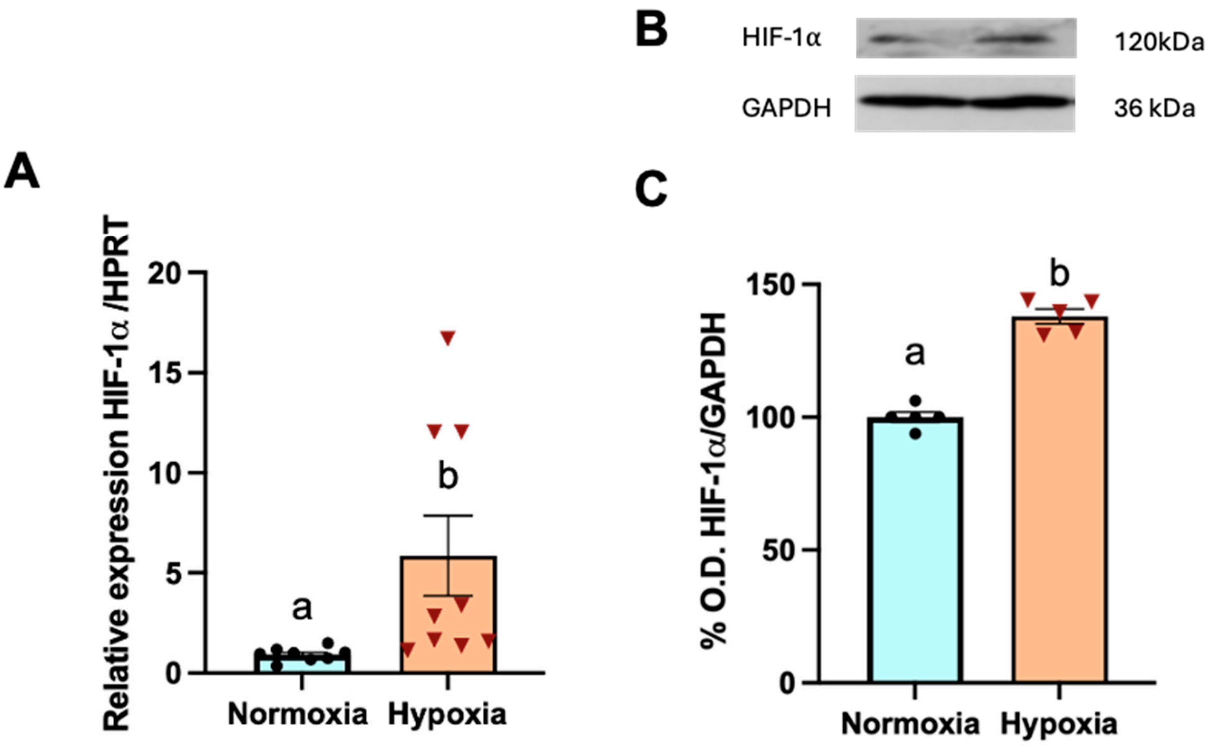

Supplementary Figure S2. Hypoxia increases the expression of HIF-1α during the acute phase in the neonatal cerebellum. Animals were exposed to hypoxic conditions for 2 hours on postnatal day 2 (P2). (A) Relative gene expression of HIF-1α mRNA (n = 7/group). (B) Western blotting of HIF-1α (120 kDa) and GAPDH (36 kDa) as loading control (n = 5/group). (C) Densitometric analysis of Western blotting. Hypoxanthine phosphoribosyl-transferase (HPRT) was used as the housekeeping gene. Results are shown as mean ± SEM. Different letters indicate significant differences between experimental groups ($p < 0.05$) compared with control normoxia determined by Student's t-test.

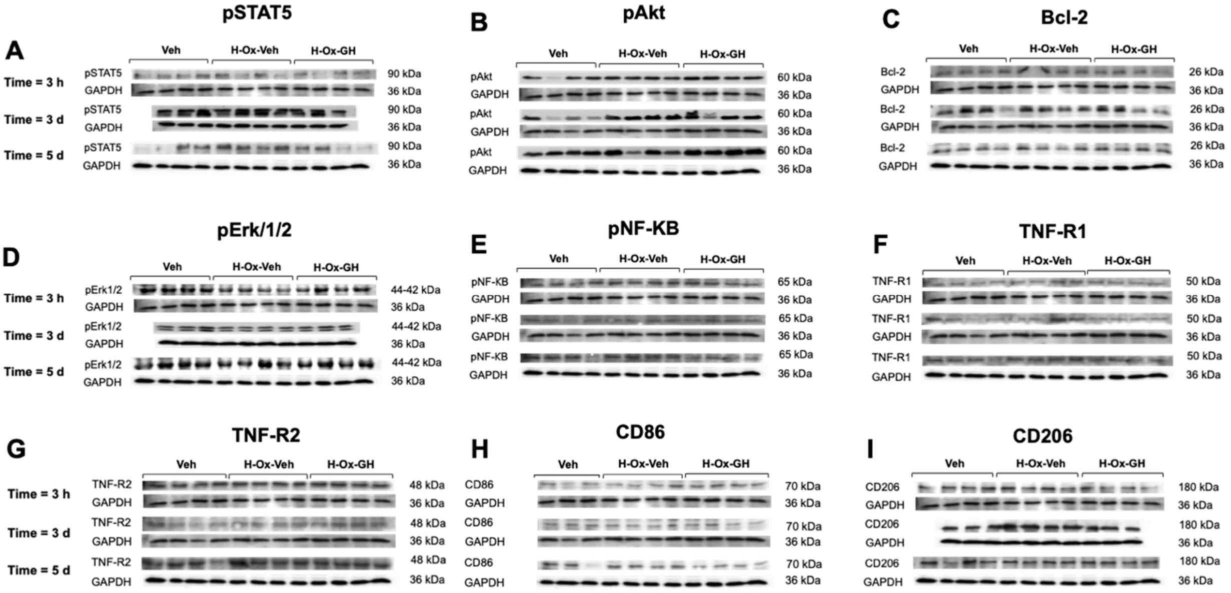

Supplementary Figure S3. Western blots showing the effect of GH treatment on signaling pathways, anti-apoptotic, and inflammatory markers during hypoxic injury in the neonatal cerebellum. Animals were exposed to hypoxia for 2 h at P2. Analyses were performed at Time = 3 h (latent phase, P2), Time = 3 d (early secondary phase, P4), and Time = 5 d (late secondary phase, P6). Proteins analyzed: (A) pStat5 (90 kDa, n = 8/group), (B) pAkt (60 kDa, n = 8/group), (C) Bcl-2 (26 kDa, n = 4/group), (D) pErk1/2 (42–44 kDa, n = 8/group), (E) pNF-κB (65 kDa, n = 8/group), (F) TNF-R1 (50 kDa, n = 4/group), (G) TNF-R2 (48 kDa, n = 4/group), (H) CD86 (70 kDa, n = 4/group), and (I) CD206 (180 kDa, n = 4/group). GAPDH (36 kDa) was used as a loading control. Groups: Vehicle (Veh), Reoxygenation (H-Ox-Veh), and bovine GH (H-Ox-GH, 0.1 mg/kg/24 h).

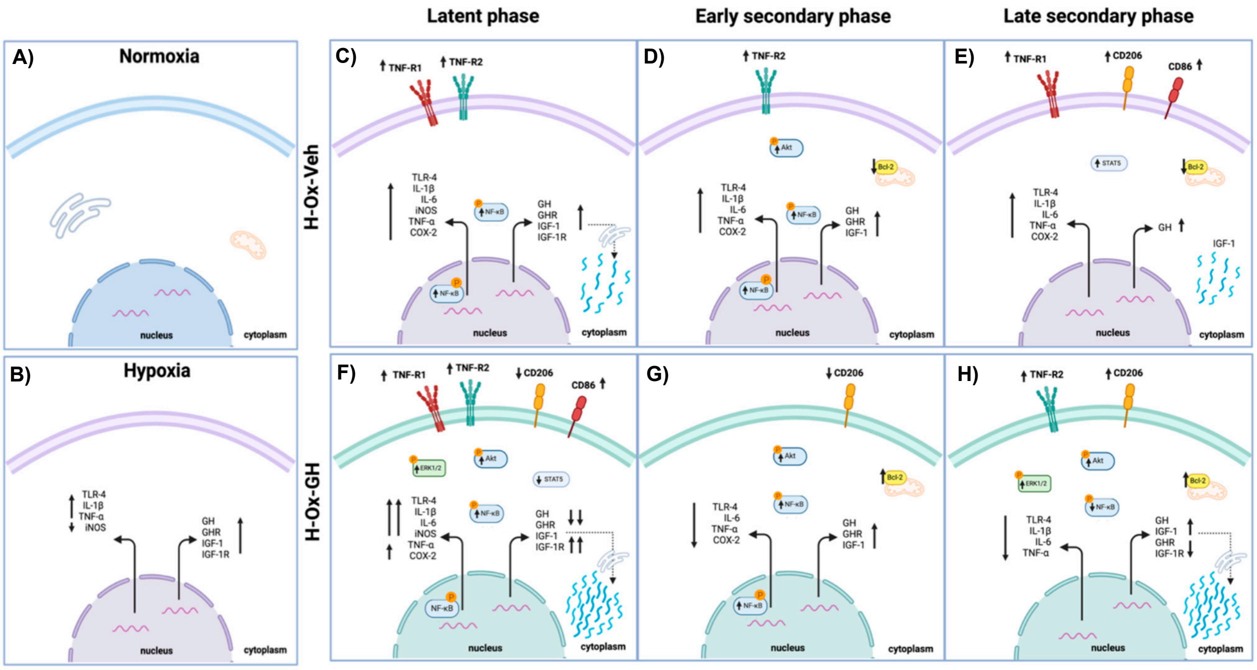

Supplementary Figure S4. Illustrative summary of the effects of GH on inflammatory markers, apoptotic factors, and intracellular signaling pathways in the neonatal cerebellum subjected to global hypoxia. This schematic represents the summarized data from Figure 2 (inflammatory markers, qPCR), Figure 3 (inflammatory and apoptotic markers, signaling pathways, WB), and Figure 4 (GH, IGF-1, and receptors, qPCR). Four distinct phases are delineated:

- Time = 0 h (acute phase, P2): hypoxia (2 h).
- Time = 3 h (latent phase, P2): 3 h post-hypoxia.
- Time = 3 d (early secondary phase, P4): 3 days post-hypoxia.
- Time = 4 d (secondary phase, P5), 4 days post-hypoxia.
- Time = 5 d (late secondary phase, P6): 5 days post-hypoxia.

Groups: (A) Normoxia, (B) Hypoxia, (C–E) Reoxygenation (H-Ox-Veh), (F–H) bovine GH (H-Ox-GH, 0.1 mg/kg/24h).

Hypoxia at Time = 0 h (acute phase, P2, B) induces a sustained inflammatory response, starting at the acute phase and characterized by increased expression of TLR-4, IL-1β, and TNF-α, as well as upregulation of GH, GHR, IGF-1, and IGF-1R.

During the latent phase (Time = 3 h, P2; C), the inflammatory response intensifies, with elevated TLR-4, IL-1β, TNF-α, IL-6, COX-2, iNOS, TNF-R1, TNF-R2, and pNF-κB, along with persistent GH, GHR, IGF-1, and IGF-1R expression.

In the early and late secondary phases (Time = 3 d, P4 and Time = 5 d, P6; D–E), inflammation persists, marked by sustained expression of pro-inflammatory mediators (TLR-4, IL-1β, TNF-α, IL-6, COX-2, iNOS, TNF-R1, CD86, and pNF-κB), concomitant with decreased pro-survival protein Bcl-2 and reduced IGF-1/IGF-1R.

GH exerts a biphasic modulatory effect. During the latent phase (Time = 3 h, P2; F), it transiently promotes a pro-inflammatory profile (↑ TLR-4, IL-1β, IL-6, iNOS, CD86, and IGF-1 protein) while activating survival pathways (TNF-R1, TNF-R2, pNF-κB, pErk1/2, Akt). In contrast, in the early and late secondary phases (Time = 3 d, P4 and Time = 5 d, P6; G–H), GH shifts to an anti-inflammatory profile, downregulating TLR-4, IL-1β, TNF-α, IL-6, and pNF-κB, while upregulating TNF-R2, pErk1/2, pAkt, Bcl-2, and CD206. Importantly, GH consistently promotes IGF-1 protein synthesis from the initial administration through all phases of treatment after hypoxic injury. The prefix p- indicates protein phosphorylation/activation; qPCR, quantitative polymerase chain reaction; WB, Western blot.

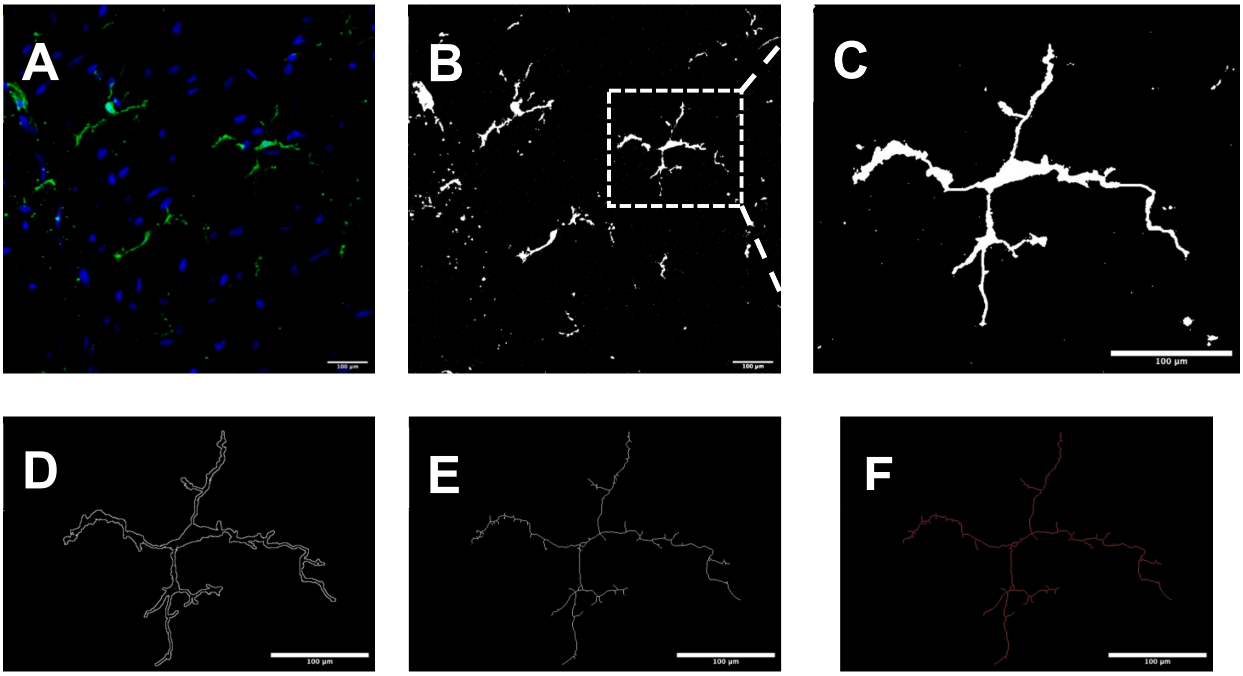

Supplementary Figure S5. Single-cell methods to assess microglial morphology in ImageJ. (A) 40X Z-stack photomicrograph of Iba1-stained microglia. (B) 40X photomicrograph of Iba1-stained microglia in binary. (C) Binary isolated microglia. (D) For fractal analysis, isolated microglia were converted to outlines and analyzed using the FracLac plugin. (E) For individual skeletal analysis, microglia were skeletonized and analyzed using the skeletal analysis plugin. (F) Enlarged version of a skeletonized cell and a tagged skeleton from images. Scale bar = 100 µm.
